# Evaluating and mitigating the potential indirect effect of COVID-19 on control programmes for seven neglected tropical diseases: a modelling study

**DOI:** 10.1016/S2214-109X(22)00360-6

**Published:** 2022-10-11

**Authors:** Anna Borlase, Epke A Le Rutte, Soledad Castaño, David J Blok, Jaspreet Toor, Federica Giardina, Emma L Davis, NTD Modelling Consortium, Maryam Aliee, Roy M Anderson, Diepreye Ayabina, Maria-Gloria Basáñez, Seth Blumberg, Rocio M Caja Rivera, Nakul Chitnis, Luc E Coffeng, Christopher N Davis, Michael Deiner, Peter J Diggle, Claudio Fronterrè, Emanuele Giorgi, Matthew Graham, Jonathan ID Hamley, T Deirdre Hollingsworth, Matt J Keeling, Klodeta Kura, Thomas M Lietman, Veronica Malizia, Graham F Medley, Edwin Michael, S Mwangi Thumbi, Nyamai Mutono, Travis Porco, Joaquín M Prada, Kat S Rock, Swarnali Sharma, Simon Spencer, Wilma A Stolk, Panayiota Touloupou, Andreia Vasconcelos, Carolin Vegvari, Sake J de Vlas

**Affiliations:** a Big Data Institute, Li Ka Shing Centre for Health Information and Discovery, University of Oxford, Oxford, UK; b Department of Public Health, Erasmus MC, University Medical Center Rotterdam, Rotterdam, Netherlands; c Department of Epidemiology and Public Health, Swiss Tropical and Public Health Institute, Allschwil, Switzerland; d University of Basel, Basel, Switzerland; e LYO-X, Allschwil, Switzerland; f MRC Centre for Global Infectious Disease Analysis, Department of Infectious Disease Epidemiology, School of Public Health, Imperial College London, London, UK; g Department of Health Evidence, Radboud University Medical Center, Nijmegen, Netherlands; h Mathematics Institute, University of Warwick, Coventry, UK

## Abstract

**Background:**

In line with movement restrictions and physical distancing essential for the control of the COVID-19 pandemic, WHO recommended postponement of all neglected tropical disease (NTD) control activities that involve community-based surveys, active case finding, and mass drug administration in April, 2020. Following revised guidance later in 2020, and after interruptions to NTD programmes of varying lengths, NTD programmes gradually restarted in the context of an ongoing pandemic. However, ongoing challenges and service gaps have been reported. This study aimed to evaluate the potential effect of the programmatic interruptions and strategies to mitigate this effect.

**Methods:**

For seven NTDs, namely soil-transmitted helminths, schistosomiasis, lymphatic filariasis, onchocerciasis, trachoma, visceral leishmaniasis, and human African trypanosomiasis, we used mathematical transmission models to simulate the effect of programme interruptions on the dynamics of each of these diseases in different endemic settings. We also explored the potential benefit of implementing mitigation strategies, primarily in terms of minimising the delays to control targets.

**Findings:**

We show that the effect of the COVID-19-induced interruption in terms of delay to achieving elimination goals might in some cases be much longer than the duration of the interruption. For schistosomiasis, onchocerciasis, trachoma, and visceral leishmaniasis, a mean delay of 2–3 years for a 1-year interruption is predicted in areas of highest prevalence. We also show that these delays can largely be mitigated by measures such as additional mass drug administration or enhanced case-finding.

**Interpretation:**

The COVID-19 pandemic has brought infectious disease control to the forefront of global consciousness. It is essential that the NTDs, so long neglected in terms of research and financial support, are not overlooked, and remain a priority in health service planning and funding.

**Funding:**

Bill & Melinda Gates Foundation, Medical Research Council, and the UK Foreign, Commonwealth & Development Office.

## Introduction

Neglected tropical diseases (NTDs) represent a diverse group of diseases (including viral, bacterial, protozoan, helminth, and fungal infections) that share the major geographical and social context of being predominantly seen in tropical or subtropical regions and in low-income communities around the world. NTDs can cause a wide range of long-term morbidities, often leading to irreversible disability, and are acknowledged to be both the drivers and manifestations of poverty, disproportionately prevalent among people who live below the World Bank poverty figure of US$1·25 per day.^1^, ^2^

In 2015–19, great progress was made towards ambitious NTD control and elimination targets set by WHO for 2020 in the first NTD roadmap,^3^ following concerted efforts and game-changing collaborations, in particular pledges from donors, pharmaceutical companies, and other stakeholders to support countries to implement strategic WHO-recommended interventions, with the aim of eliminating the enormous public health burden of NTDs.^4^, ^5^

In response to the COVID-19 pandemic, on April 1, 2020, WHO announced interim guidance recommending that NTD control programmes postpone all activities relating to active case detection, community-based surveys, and mass drug administration.^6^ In July, 2020, this recommendation was then followed by revised interim guidance from WHO for restarting NTD programmes; ongoing advice, guidance, and training from WHO and others has aimed to support NTD programmes in the context of an evolving pandemic, focusing on risk–benefit analyses and implementation of precautionary measures to minimise the spread of SARS-CoV-2 (such as physical distancing and the use of personal protective equipment).^7^, ^8^

**Research in context**

**Evidence before this study**

We searched PubMed and Science Direct for studies considering the effect of the COVID-19 pandemic on neglected tropical diseases (NTDs) in general using the search terms “neglected tropical diseases” AND “COVID-19”, with no language or date restrictions. We also searched for studies examining the effect of the pandemic on each of the seven diseases considered in this study individually (“soil transmitted helminths” AND “COVID-19”/ “schistosomiasis” AND “COVID-19”/ “onchocerciasis” AND “COVID-19”/ “lymphatic filariasis” AND “COVID-19”/ “trachoma” AND “COVID-19”/ “visceral leishmaniasis” AND “COVID-19”/ “human African trypanosomiasis” AND “COVID-19”). We also consulted the WHO website and websites for NTD programme funders. Articles were considered relevant if they considered the effect of COVID-19-induced disruptions to NTD control. Several articles described the challenges of restarting programmes in the context of COVID-19, and some commentaries described concerns within the NTD community regarding how the COVID-19 pandemic diverted much needed financial and human resources, and predicted that recent progress might be reverted, with emphasis placed on the fragility of health systems and economies in many countries where NTDs are endemic. We did not find any empirical studies examining or quantifying the public health consequences of disruption to NTD control programmes in terms of increases in infection prevalence or incidence, or excess morbidity or mortality.

**Added value of this study**

Using previously validated mechanistic transmission models, this study provides quantitative insights into the potential effect of the COVID-19 pandemic in terms of delays to achieving control and elimination targets and how these might be mitigated. Given the scarcity of empirical data and the fact that NTD surveillance has also been severely disrupted by the pandemic, the modelling approach is one of the few ways in which the effect of disruption to NTD programmes can be evaluated in a timely manner such that prioritisation of resources and planning might be able to mitigate this effect. Our study enables broad comparison, both between diseases and between endemic settings for each disease, providing much needed guidance regarding where attention and resources should be focused as the world learns to live with COVID-19.

**Implications of all the available evidence**

This study highlights the need to avoid overlooking the indirect consequences for control and elimination of NTDs caused by the COVID-19 pandemic. The underlying dynamics of each NTD and the level of endemicity in each setting will influence the rate of resurgence, with high transmission areas and diseases with the fastest bounce-back rate presenting the greatest challenges. By implementing appropriate mitigation strategies, the long-term public health effect of the COVID-19 pandemic on efforts to control NTDs could be effectively minimised, and NTD programmes should receive the necessary support to facilitate such measures in a timely way. Furthermore, leveraging the currently raised awareness of infectious disease control could potentially galvanise efforts, and more intensive remedial strategies might enable acceleration of progress towards the ultimate control and elimination goals for the NTDs.

Guinea was reported to be one of the first countries to resume mass drug administration after an interruption of approximately 6 months and, despite an entirely changed public health landscape, in most NTD endemic countries there has been a gradual and progressive resumption of community-based NTD interventions following interruptions to activities of varying durations.^9^, ^10^, ^11^ However, substantial challenges and service gaps have been reported, including missed rounds of mass drug administration and vector control (eg, indoor residual spraying of insecticide) in 2020; hesitancy or refusal of communities to participate; reassignment of NTD programme personnel to support COVID-19 interventions; delays in active case finding and presentation of cases to health facilities (passive case detection); delays in manufacture and supply of NTD medicines; and discontinuation of monitoring and evaluation activities, including population-based surveys.^10^, ^11^, ^12^, ^13^, ^14^ The 2021 national pulse survey, carried out by WHO on continuity of essential health services during the COVID-19 pandemic, found that the proportion of countries reporting severe disruptions to NTD activities was the highest of all health services, with mass drug administration reported to be both the most frequently and most severely affected of all NTD services.^12^ This finding raises serious concerns that the indirect effect of the COVID-19 pandemic could lead to substantial losses to many of the achievements of recent years (eg, the ten-fold reductions in incidence of human African trypanosomiasis since 2013 and the five-fold reductions in incidence of visceral leishmaniasis since 2011, both diseases that require case-finding and disease management), and threaten progress towards the 2021–30 targets proposed by the second WHO roadmap on NTDs.^5^, ^15^, ^16^

Previous interruptions to public health programmes have had unexpected and profound consequences. For example, the number of direct Ebola virus-related deaths during the 2013–16 outbreak in west Africa was most likely exceeded by the number of indirect deaths caused by interruption of routine health activities, changes in health-seeking behaviour, and diversion of scarce health resources.^17^, ^18^ The implications of the Ebola virus outbreak for NTD programmes was generally not well quantified but, as an example, up to a 10-times increase in disability-adjusted life-years generated was estimated as a consequence of the Ebola virus-related scale-back of human African trypanosomiasis programmes in Guinea.^19^

Although some NTDs can be fatal within a relatively short timeframe, the most severe sequelae are more generally seen after many years of chronic or repeated infection. In contrast to many of the vaccine-preventable illnesses, NTDs are not typically associated with large outbreaks and catastrophic peaks in mortality, seen for example with lapses in measles vaccination coverage.^20^ The implications of the long timecourse for the morbidity associated with many NTDs is that the public health consequences of any programmatic interruption due to the COVID-19 pandemic might not become apparent for many years, particularly given that surveillance activities have also been disrupted. However, this also provides an opportunity to mitigate the effect of programmatic disruption, if timely and decisive action is taken.

In the third year of the pandemic, the following questions are priorities for planning, resource allocation and advocacy. What will be the effect of disruptions to NTD programmes due to the COVID-19 pandemic? In which communities is the effect likely to be greatest? And, crucially, what can be done to mitigate the effect?

Focusing on seven NTDs (soil-transmitted helminths, schistosomiasis, lymphatic filariasis, onchocerciasis, trachoma, visceral leishmaniasis, and gambiense human African trypanosomiasis), we aimed to address these priority questions. Whereas previous modelling studies examining the potential effect of COVID-19 on NTD programmes have focused on single diseases, our multidisease, multimodel study aimed to provide broad comparison, both between diseases and between endemic settings for each disease, to provide guidance regarding where attention and resources should be focused as the world learns to live with COVID-19, and to support a technical report published by WHO focusing on the same priority questions.^21^

Using well established transmission models, and with output intended to be applicable to a range of endemic settings, we evaluated the potential effect of the disruptions caused by the COVID-19 pandemic on control programmes in terms of the potential delay to reaching the control and elimination targets set by WHO, and explored the possible benefits of mitigation strategies in the years following resumption of activities for each disease.

## Methods

### Study design

In our modelling study, we considered seven NTDs and selected existing well established mathematical transmission models developed under the NTD Modelling Consortium (Table 1, Table 2). For soil-transmitted helminths, lymphatic filariasis, onchocerciasis, and trachoma, stochastic models were used, and for schistosomiasis, visceral leishmaniasis, and gambiense human African trypanosomiasis, the models were deterministic. All models are described in detail in the appendix (pp 5–31).

**Table 1:** Summary of the seven NTDs modelled in this study

	**Models used**	Causative agents*	**Mode of transmission**	**Main clinical manifestations in endemic settings**	**Number of people affected worldwide**	**2030 target: EPHP or EOT**
Soil-transmitted helminths	Erasmus MC;^22^, ^23^, ^24^ Imperial College London^25^	Roundworm (*Ascaris lumbricoides*), whipworm (*Trichuris trichiura*), hookworms (*Necator americanus* and *Ancylostoma duodenale*)	Primarily faecal–oral transmission (also skin penetration of larvae in hookworm)	Diarrhoea, abdominal pain, stunting, general malaise, and weakness	Approximately 1·5 billion people are infected worldwide^26^	EPHP: ≤2% prevalence of moderate-to-heavy-intensity infections among school-aged children
Schistosomiasis	Imperial College London;^27^ Oxford and SCHISTOX;^28^ only for intestinal schistosomiasis in sub-Saharan Africa	Parasitic worm, *Schistosoma mansoni*	Transmission via contact with water containing aquatic snail intermediate host	Anaemia, stunting, diarrhoea, hepatomegaly, portal hypertension, and periportal fibrosis	229 million school-aged children and adults requiring preventive chemotherapy^26^	EPHP: ≤1% prevalence of heavy-intensity infections among school-aged children
Lymphatic filariasis	Warwick (TRANSFIL);^29^, ^30^ Erasmus MC, LYMFASIM^31^ and University of South Florida, and EPIFIL^32^	Parasitic worm, *Wuchereria bancrofti*	Mosquito vector	Hydrocele (scrotal swelling), acute lymphoedema, and chronic lymphoedema (elephantiasis) affecting limbs and genitals	Affects over 120 million people worldwide, with 593 million at risk, and is a leading cause of permanent disability^26^	EPHP: defined as passing three transmission assessment surveys; operationally equivalent to 1% prevalence of infective stages (microfilariae)
Onchocerciasis	Erasmus MC (ONCHOSIM); 33, 34 Imperial College London, and EPIONCHO^35^	Parasitic worm, *Onchocerca volvulus*	Blackfly vector	Skin disease (eg, severe itching), visual Impairment, and permanent blindness	2·9 million *O volvulus* infections worldwide^26^	EOT
Trachoma	Oxford^36^	Bacterial eye infection, *Chlamydia trachomatis*	Predominantly direct personal contact	Repeated episodes of conjunctivitis, which can lead to scarring, entropion (in-turning of eyelids), trichiasis (abrasion of cornea by the eyelashes), and eventual blindness	Responsible for blindness or visual impairment of 1·9 million people; 137 million at risk of blindness^26^	EPHP: reduction of trachomatous inflammation—follicular in children aged 1–9 years to less than 5%; prevalence of trachomatous trichiasis unknown to health system <0·2% (trichiasis not modelled here)
Visceral leishmaniasis	Erasmus MC;^37^, ^38^ only for India	Protozoa, *Leishmania donovani*	Sandfly vector	Persistent fever, enlarged liver and spleen, death if untreated	50 000–90 000 new cases per year globally^26^	EPHP: <1 per 10 000 inhabitants per year at subdistrict level in India
Gambiense human African trypanosomiasis	Warwick;^39^, ^40^ Swiss TPH;^41^ specified for DR Congo	Protozoa, *Trypanosoma brucei gambiense*	Tsetse fly vector	Stage 1: mild symptoms (eg, fever and headaches); stage 2: neurological disorders resulting in death if untreated	51 million people estimated to be at risk of infection^26^	EPHP: <1 case per 10 000 inhabitants per year averaged over a 5-year period by 2020; EOT: zero reported cases by 2030

**Figure 1:**
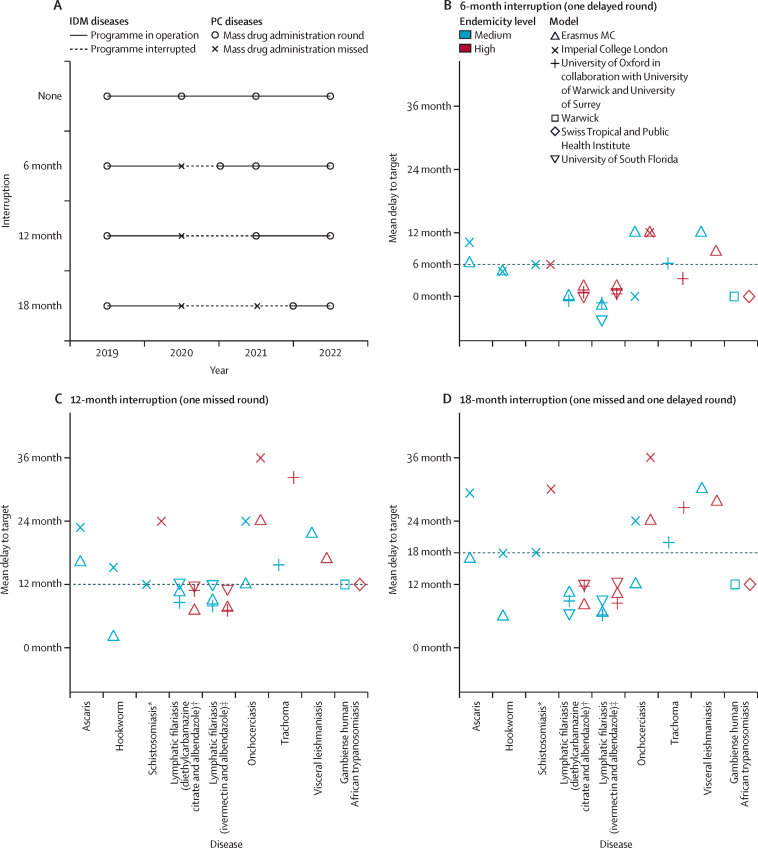
Mean delays to achieving the control or elimination targets caused by interruption to control programmes across all diseases in medium-endemic and high-endemic settings (A) Schematic of the three different scenarios. (B) 6-month interruption (one delayed round). (C) 12-month interruption (one missed round). (D) 18-month interruption (one missed and one delayed round). Control or elimination targets are as given in table 1. Medium and high endemicity are as defined in tables 3 and 4 for each disease. IDM=integrated disease management. PC=preventive chemotherapy. *Schistosoma mansoni with a low burden in adults. †Annual diethylcarbamazine citrate and albendazole treatment; dominated transmission by *Culex* mosquitoes. ‡Lymphatic filariasis with annual ivermectin and albendazole treatment; dominated transmission by *Anopheles* mosquitoes.

Further details on transmission, epidemiology, control strategies, and elimination targets for each disease are available in the WHO NTD factsheets^26^ and the WHO 2021–30 NTD roadmap.^5^ For more information on the models used see figure 1 and the appendix (pp 5–33). NTD=neglected tropical disease. EPHP=elimination as a public health problem. EOT=elimination of transmission. SCHISTOX=schistosomiasis Oxford model. TRANSFIL=transmission model of filariasis. LYMFASIM=lymphatic filariasis simulation model. EPIFIL=epidemiological model of filariasis. ONCHOSIM=onchocerciasis simulation model. EPIONCHO=epidemiological model of onchocerciasis.

* Causative agents considered in this study.

**Table 2:** Summary of control and mitigation measures modelled for each of the seven NTDs

	**WHO-recommended control method**	**Transmission control strategy modelled**	**Mitigation strategy modelled**
Soil-transmitted helminths	Annual or semi-annual school-based MDA of albendazole and mebendazole (school-aged children only)	Annual or semi-annual school-based MDA of albendazole and mebendazole in preschool-aged children and school-aged children (aged 2–15 years) at 75% coverage	1 year of enhanced (community-wide) MDA at 75% coverage
Schistosomiasis	Annual MDA with praziquantel at 75% coverage in either all age groups from age 2 years, or test and treat dependent on prevalence (before 2022 annual school-based MDA for school-aged children only)	Annual school-based MDA of praziquantel in school-aged children (aged 5–14 years) at 75% coverage	1 year of enhanced (community-wide) MDA at 85% school-aged children and 40% adult coverage
Lymphatic filariasis	Biannual albendazole (areas coendemic with loiasis); annual ivermectin with albendazole (areas with onchocerciasis); diethylcarbamazine citrate and albendazole (areas without onchocerciasis)	Annual community-based MDA with a combination of either ivermectin or diethylcarbamazine citrate with albendazole: ivermectin with albendazole if coendemic with loa loa, otherwise either diethylcarbamazine citrate with albendazole, or ivermectin with diethylcarbamazine citrate and albendazole; 65% coverage	An additional community-based MDA in the year following the interruption at 65% coverage
Onchocerciasis	Annual community-based MDA with ivermectin	Annual community-based MDA with ivermectin; 65% coverage of the total population and 5% systematic non-participation	An additional community-based MDA in the year following the interruption at 65% coverage
Trachoma	Annual community-based MDA with azithromycin	Annual community-based MDA with azithromycin at 80% coverage	An additional community-based MDA round in the year following restart at 80% coverage (2 rounds delivered 6 months apart)
Visceral leishmaniasis	Vector control through indoor residual spraying and active case detection followed by free treatment	Attack phase (5 years): indoor residual spraying assumed to reduce sandflies by 67% and active case detection reduces time to case detection by 25%; consolidation phase: indoor residual spraying decreased to 45% but active case detection increased, reducing time to case detection by 50%	If interruption occurs during attack phase, this is then extended by the same length as the interruption; if interruption occurs during consolidation phase, a temporary attack phase equivalent to length of interruption is initiated
Gambiense human African trypanosomiasis	Active case detection (mobile teams) and passive surveillance	Active case detection (mobile teams) and passive surveillance	Resuming active screening at maximum historical level with passive detection set back to values before interruption

Further details on transmission, epidemiology, control strategies, and elimination targets for each disease are available in WHO NTD factsheets^26^ and the WHO 2021–30 NTD roadmap.^5^ NTD=neglected tropical disease. MDA=mass drug administration.

Key aspects regarding each of the seven diseases, including control and elimination targets set by WHO, are summarised in table 1. The seven diseases can be divided into those for which control interventions primarily focus on preventive chemotherapy, generally delivered as mass drug administration (ie, soil-transmitted helminths, schistosomiasis, lymphatic filariasis, onchocerciasis, and trachoma) and those for which intensive disease management is the cornerstone of control (ie, visceral leishmaniasis [focusing on the Indian subcontinent], and gambiense human African trypanosomiasis).

### Setting

We simulated at least two generalised settings for each disease, representing high and medium levels of precontrol endemicity (definitions of high and medium endemicity levels for each disease are specified in tables 3 and 4). The baseline level of endemicity for a given community (ie, infection prevalence or incidence before implementation of any large-scale interventions) might be determined by a wide range of factors, including vector population density, access to clean water, and types of housing. The key factors that varied for each disease to represent differing settings are described in the appendix (pp 5–31).

### Procedures

We compared the projected timelines towards the 2030 goals (had disruption due to COVID-19 not occurred) to simulated periods of programmatic interruption for each disease in both high and medium endemic settings. We considered 6-month, 12-month, and 18-month interruptions to mass drug administration (preventive chemotherapy diseases) or active case finding (intensive disease management diseases). The simulated interruptions and restart timings are represented schematically in figure 1.

We also considered the effect of mitigation strategies implemented in the year following a 12-month interruption, selecting an appropriate mitigation strategy for each disease. Baseline control and mitigation measures modelled for each disease are summarised in table 2.

### Role of the funding source

A representative of the funding sources was involved in initial discussions regarding conceptualising and planning the study and gave feedback on preliminary results. The funders of the study had no role in study design, model implementation, analysis, or writing of the manuscript.

## Results

For the majority of the diseases and settings considered here, the mean delay to achieving the 2030 target is similar to the assumed length of interruption to the control programme (figure 1, tables 3, 4). However, for onchocerciasis and visceral leishmaniasis, the delay to the target is expected to be substantially longer than the interruption (up to a 3-year mean delay for a 1-year interruption). Similarly, if the interruption is 12 months or longer, in both high-endemic trachoma and high-endemic schistosomiasis settings, the delay is expected to be much longer than the duration of the interruption. However, if appropriate mitigation strategies are implemented (Table 3, Table 4), reaching the 2030 goals (or for those diseases for which reaching the 2030 targets is unlikely under current strategies, reaching the same prevalence level expected by 2030 if there had been no interruption) should be achievable for all diseases. For broad comparison, the bounce-back rates for each disease following interruption are also described in the appendix (p 4). Further disease-specific results, results from alternative models, and exploration of uncertainties regarding model assumptions are detailed in the appendix (pp 5–31).

**Table 3:** Timeline (mean years) to 2030 target for the preventive chemotherapy diseases for no interruption, a 12-month interruption, and a 12-month interruption with an example mitigation strategy

	**Target**	**Mitigation strategy**	**Preventive chemotherapy diseases (endemicity or baseline prevalence)**	**Years to target with no interruption**	**Years to target with 12-month interruption**	**Years to target with 12-month interruption and mitigation**
Hookworm infection	Elimination as a public health problem (≤2% prevalence of moderate-to-heavy-intensity infections of >2000 epg in school-aged children)	1 year of enhanced MDA (community-wide rather than in school-aged children only)	Medium (20–50%) prevalence in school-aged children	8·1	8·3	6·8
Ascariasis	Elimination as a public health problem (≤2% prevalence of moderate-to-heavy-intensity infections of >5000 epg in school-aged children)	1 year of enhanced MDA (community-wide rather than in school-aged children only)	Medium (20–50%) prevalence in school-aged children	6·4	7·7	3·9
Schistosomiasis (*Schistosoma mansoni*)	Elimination as a public health problem (≤1% prevalence of heavy-intensity infections of ≥400 epg in school-aged children)	1 year of enhanced MDA (community-wide rather than in school-aged children only)	High (70% in school-aged children) with either low adult burden or high adult burden; medium (30% in school-aged children) with either low adult burden or high adult burden	High: 7·0 in school-aged children with low adult burden or target predicted to not be achieved by 2030 with high adult burden; medium: 2·0 in school-aged children with low adult burden or 3·0 with high adult burden	High: 9·0 in school-aged children with low adult burden or target predicted to not be achieved by 2030 with high adult burden; medium: 3·0 in school-aged children with low adult burden or 4·0 with high adult burden	High: 8·0 in school-aged children with low adult burden or target predicted to not be achieved by 2030 with high adult burden; medium: 3·0 in school-aged children with low adult burden or 0·0 with high adult burden
Lymphatic filariasis (*Wuchereria bancrofti*, ivermectin with albendazole settings in rural Africa, transmitted by *Anopheles* mosquitoes)	Elimination as a public health problem (<1% prevalence of microfilaria)	One additional round of MDA	High (15–20% prevalence); medium (5–10% prevalence)	11·4 (high); 7·7 (medium)	12·0 (high); 8·4 (medium)	11·5 (high); 7·6 (medium)
Onchocerciasis (years to catch up to 2030 prevalence of microfilaria levels without interruption)*	Elimination of transmission	One additional round of MDA	High and medium	Not predicted to be achieved by 2030	2·0–3·0 (high); 1·0–2·0 (medium)	1·0 (high); 0·0 (medium)
Trachoma	5% prevalence of TF in children aged 1–9 years (TF_1–9_ <5%)	One additional community-wide round of MDA	High (40% TF_1–9_); medium (20% TF_1–9_)	4·4 (high); 2·7 (medium)	7·1 (high); 4·0 (medium)	5·3 (high); 3·8 (medium)

epg=eggs per gram. MDA=mass drug administration. TF=trachomatous inflammation–follicular.

* Prevalence of microfilaria levels achieved in 2030 using annual MDA since 2014 were used as a reference to calculate delay in progress towards elimination of transmission due to the COVID-19 pandemic. The modelled prevalence of microfilaria levels in 2030 without interruption are 8·6% (high) and 2·0% (medium).

**Table 4:** Timeline (mean years) to the elimination target for the intensive disease management diseases for no interruption, a 12-month interruption, and a 12-month interruption with an example mitigation strategy

	**Target**	**Mitigation strategy**	**Intensive disease management diseases (baseline endemicity)**	**Years to target with no interruption**	**Years to target with 12-month interruption**	**Years to target with 12-month interruption and mitigation**
Visceral leishmaniasis	<1 visceral leishmaniasis case per 10 000 per year at subdistrict level (India)	A 12-month extension of the attack phase (increased indoor residual spraying and active case detection)	High (10 cases per 10 000 per year); medium (5 cases per 10 000 per year)	9·5 (high); 2·3 (medium)	10·9 (high); 4·1 (medium)	10·0 (high); 4·1 (medium); target reached before start of mitigation strategy
Gambiense human African trypanosomiasis	Elimination of transmission	Resuming active screening at maximum historical level with passive detection set back to values before interruption	High*; medium*	32 (high); 13 (medium)	33 (high); 14 (medium)	31 (high); 12 (medium)

The number of years to target are calculated from 2018 onwards.

* Transmission levels inferred from number of reported cases (appendix pp 22–24).

**Figure 2:**
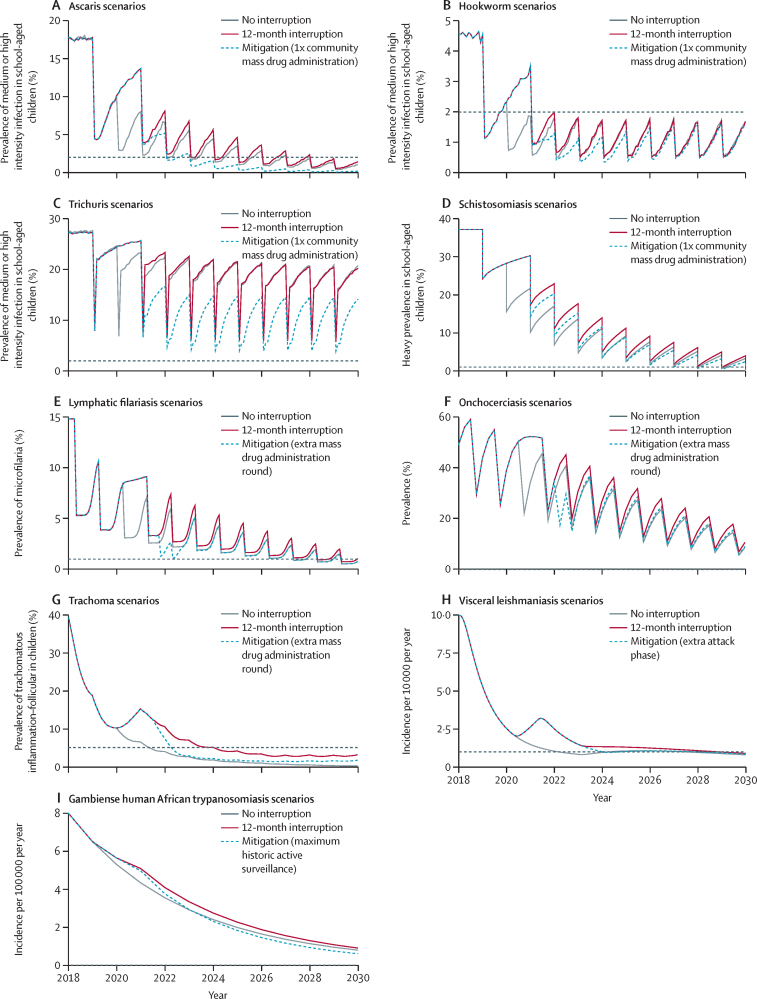
Timeline towards control and elimination targets for all diseases Scenarios with no interruption, scenarios with a 12-month interruption and no mitigation, and scenarios with a 12-month interruption with mitigation strategy modelled (table 2). Grey dashed lines represent the control or elimination target thresholds (or equivalent) for each disease. Plots represent model output for high-endemic settings for onchocerciasis, lymphatic filariasis, trachoma, and visceral leishmaniasis, high-endemic setting with high adult burden setting for schistosomiasis, and medium-endemic settings for gambiense human African trypanosomiasis and soil-transmitted helminths (ascaris, hookworm, and trichuris).

The soil-transmitted helminths considered here are those caused by intestinal nematodes, specifically *Ascaris lumbricoides*, *Trichuris trichiura*, and hookworms (*Necator americanus* and *Ancylostoma duodenale*). The 2030 goal for soil-transmitted helminths is elimination as a public health problem (EPHP), defined as reaching a prevalence of 2% or less of moderate-to-high-intensity infections in school-aged children (aged 5–14 years).^26^, ^42^ On average, in medium-endemic settings, the expected delay to achieving the 2030 goals is similar to the length of the interruption for infections caused by *A lumbricoides* and hookworm (figure 1). In high-endemic settings and those with *T trichiura* as the predominant species, the probability of reaching the EPHP goal by 2030 (regardless of any interruption) was very low (table 3). Simulations indicated that a mitigation round of community-wide mass drug administration (ie, for all ages and not only school-aged children) when programmes resume would be sufficient to compensate for a 12-month interruption in both ascariasis and hookworm infection (figure 2, table 3).

The 2030 goal for schistosomiasis is EPHP, which is defined as a reduction of heavy-intensity prevalence in school-aged children to less than 1%.^5^, ^26^ For *Schistosoma mansoni*, the causative agent for intestinal schistosomiasis modelled here, the effect of the delay, and the appropriate mitigation strategy depend both on the baseline prevalence before implementation of mass drug administration (with praziquantel) and the burden of infection in adults relative to school-aged children. A 1-year interruption in mass drug administration is predicted to lead to the EPHP goal being delayed by up to 2 years (figure 1, table 3). High-prevalence settings with a high burden in the adult population might not reach EPHP by 2030 regardless of the postponement (table 3), and in such settings adults should be treated as well as school-aged children if EPHP is to be achieved. To mitigate the delay and accelerate progress once schistosomiasis programmes can resume, it is important that surveys are carried out to collect infection metrics in school-aged children and adults, particularly in high-prevalence settings, as this will be needed to determine whether treatment of adults in the population is also required (figure 2) and what the optimal coverage levels should be.

The 2030 goal for lymphatic filariasis is EPHP, operationally equivalent to reaching 1% microfilaria prevalence.^5^, ^26^ The mean delay to achieving this target is estimated to be similar to or less than the interruption length for all modelled scenarios (figure 1)—ie, missing 1 year of mass drug administration (with ivermectin and albendazole in Africa) is expected to result in an average delay of 1 year or less.

The 2030 WHO target for onchocerciasis is elimination of transmission (EOT). Therefore, no fixed prevalence thresholds associated with true elimination are used, because these are likely to differ by level of initial endemicity.^31^ Country-wide EOT is targeted for 2030 in 12 countries, whereas other countries are expected to achieve this in one or more foci within the country.^5^ The effect of a 1-year interruption to annual mass drug administration (with ivermectin) would result in an immediate increase of microfilarial prevalence in the following year. Ivermectin is only partially macrofilaricidal (ie, does not kill adult worms rapidly), and therefore, microfilarial dynamics are important. It is estimated that this increase in microfilaria prevalence would delay the reductions that would be achieved in 2030 by 1–2 years in medium-endemic settings and 2–3 years in high-endemic settings (figure 2). Increasing the frequency of ivermectin mass drug administration to biannual with the same coverage (65%) during the year following a 12-month interruption would reduce the delay to 0 years in medium-endemic settings and to 1 year in high-endemic settings (figure 2, table 1). In high-endemic areas, if the same microfilaria prevalence level as anticipated for 2030 without interruption is to be achieved, biannual mass drug administration should be implemented for an additional year.

The primary EPHP target for trachoma is a prevalence of trachomatous inflammation–follicular of less than 5% in children aged 1–9 years. Before the interruption caused by the COVID-19 pandemic, the majority of formerly endemic countries were projected to reach this EPHP threshold by 2030 with annual community-wide mass drug administration using oral azithromycin (or topical tetracycline in infants).^43^ Missing a single round of mass drug administration in high-endemic settings is predicted to lead to an estimated mean delay of 2·7 years to reach the EPHP threshold (figure 1, table 3), compared with medium-endemic settings where the delay is roughly equivalent to the length of the interruption. However, in high-endemic settings, implementing the mitigation strategy of an additional round of community-wide mass drug administration in the year following a 12-month interruption decreases the predicted delay in reaching the EPHP target to just under a year.

Visceral leishmaniasis (caused by a protozoan parasite transmitted by sandflies) is targeted for EPHP in India where it is anthroponotic and caused by *Leishmania donovani*. EPHP is defined as less than one visceral leishmaniasis case per 10 000 population per year at subdistrict level. Interventions comprise vector control through indoor residual spraying of insecticide and active-case detection followed by free treatment. Control starts with a 5-year attack phase of intense indoor residual spraying and active case detection, followed by a consolidation phase, with reduced coverage of indoor residual spraying and intensified active-case detection. We simulated interruptions to indoor residual spraying and active case detection, but assumed passive case detection followed by treatment was still in place during simulated interruptions. A 12-month interruption during the attack phase of the control programme is estimated to cause a delay in reaching the target of approximately 1·5 years in high-endemic settings and approximately 2 years in medium-endemic settings (figure 1, table 4). An extended duration of the attack phase (of equivalent length to the duration of the interruption) is anticipated to reduce the delay to approximately 6 months in previously high-endemic settings (figure 2, table 4), whereas in moderately endemic settings such a mitigation strategy has little additional effect.

Gambiense human African trypanosomiasis (caused by a protozoan parasite transmitted by tsetse flies) is targeted for EOT by 2030^5^ with zero reported cases given as a proxy measure for achievement of the goal. Sustained active screening in addition to passive surveillance are core control activities in the management of this disease in many regions including the Democratic Republic of the Congo, the country with the highest burden of gambiense human African trypanosomiasis cases. In addition to active screening being interrupted in the Democratic Republic of the Congo, passive surveillance might also have been affected. Gambiense human African trypanosomiasis is a disease with slow progression and, as such, the effect of interruption to control activities due to the COVID-19 pandemic for the time periods explored here (6–18 months) is anticipated to be minimal in medium-risk settings, with EOT delayed by a similar timescale to the length of the interruption (figure 1, table 4). Results suggest that retaining partially functioning passive surveillance can help to prevent substantial (temporary) increases in mortality. Furthermore, high-risk settings might have already required intensified interventions to meet EOT by 2030, and a strong postpandemic response (ie, resuming active screening at historical highest levels for sustained periods) could not only mitigate the delays to the target in medium-risk regions (figure 2) but accelerate progress in reducing transmission in high-endemic areas where the 2030 EOT target is unlikely to be reached.

## Discussion

The novel threat of the COVID-19 pandemic has brought unprecedented global attention to infectious disease control, and yet there is growing concern that if attention is diverted for too long from the ongoing burden of suffering caused by NTDs, many of the gains made since the adoption of the 2011 WHO roadmap could be largely undone.^15^, ^44^

Using disease-specific models for seven NTDs, we have shown that the greatest effect of the interruption to community-based NTD control activities due to the COVID-19 crisis is likely to be seen in communities with highest endemicity. A 1-year delay might, in the case of schistosomiasis, onchocerciasis, trachoma, and visceral leishmaniasis, translate into disproportionately long delays in reaching control targets (EPHP or EOT). However, we have also shown through modelling disease-specific mitigation strategies, including enhanced mass drug administration, or reimplementation of intense control measures, that these delays can be effectively minimised in most cases.

We have focused on impact in terms of timeline to achieving elimination targets, with such targets representing an important focus for advocacy, planning, and monitoring. However, for many of the NTDs there is a complex relationship, and often a temporal disconnect between the underlying dynamics of infection and long-term pathology (table 1), meaning that the true public health effects of programmatic interruptions, in terms of morbidity, are much harder to quantify. It is important to note that any delay to achieving targets will in most cases also equate to an increased burden of morbidity, due to increased incidence or duration of infections, or worm burden in the case of helminth infections, with the most severe and disabling consequences of NTDs generally seen with prolonged, repeated, or heavy infection burden. For example, in the case of visceral leishmaniasis, a short-term peak in cases will most likely lead to a peak in post-kala-azar dermal leishmaniasis (a sequela of visceral leishmaniasis) in 1–5 years.^45^ In the case of trachoma, even if EPHP thresholds are met before 2030, any surge in transmission after interruption of mass drug administration will translate into more cases of infection, meaning greater accumulation of the damage caused by repeated infections, which ultimately leads to blindness, often decades later.^46^ For onchocerciasis and lymphatic filariasis, for which predicted delay to the goal might be small, any additional burden of transmission and morbidity caused by the interruption is likely to be concentrated in individuals with existing infection (lymphatic filariasis) or in young children (onchocerciasis) due to underlying heterogeneities in exposure patterns and risk factors.^47^ For lymphatic filariasis, the long-term implications of this additional burden of infection might include lymphatic, tissue, and organ damage, and an increase in acute and chronic lymphoedema, which can lead to permanent disability.^48^ For onchocerciasis, the additional burden in some individuals can lead to an increase in acute dermatological signs, such as acute papular onchodermatitis, and ocular signs including punctate keratitis, whereas increased infection in young children could lead to onchocerciasis-associated epilepsy later in life.^49^, ^50^ Incorporating morbidity and mortality into dynamic models for all NTDs is a research priority and should be a focus for future work.

To minimise the long-term effect on morbidity, any mitigation strategies should be implemented as soon as is practicable. However, it is acknowledged that there will be substantial challenges to implementing the mitigation strategies that we have modelled, and many studies aiming to evaluate the challenges of programmatic restarts have emphasised the increased costs associated with effectively delivering community-based NTD activities in the context of COVID-19.^10^, ^13^, ^14^, ^51^, ^52^, ^53^, ^54^ Reasons given for increased costs include personal protective equipment, the need for enhanced training, time taken for sensitisation to overcome hesitancy or refusals due to the misinformation about the pandemic, and increased costs associated with delivering mass drug administration door to door (rather than community delivery at a central distribution point, or schools-based delivery for soil-transmitted helminths and schistosomiasis).

The increased time and financial cost of NTD activities in the context of COVID-19 are expected to vary considerably, but overall cost increases of around 30% for NTD programmes have been estimated.^10^, ^53^, ^55^ This is particularly concerning given the economic devastation caused by the pandemic, which now disproportionately affects the economics of some of the world's most endemic NTD countries, with an estimated 100 million additional people now living in extreme poverty compared with 2019.^44^, ^56^ Our results show the need to prioritise restarting NTD programmes, and meeting the increased costs of delivering programmes in the context of COVID-19 (in particular, costs associated with any mitigation strategies) should be considered a funding priority.

There are several disease settings where our models predicted that under current control measures control elimination goals would not be achieved before 2030, even without the disruption caused by the pandemic. These include high-prevalence soil-transmitted helminths settings, high-prevalence *S mansoni* settings where burden is also high in adults, high-prevalence onchocerciasis settings (with high vector biting rates), and high-endemic gambiense human African trypanosomiasis settings. In some cases, the models predicted that the implementation of an enhanced protocol for 1 or more years, as a post-COVID-19 mitigation strategy, would actually accelerate the progress towards elimination (including ascariasis and gambiense human African trypanosomiasis). Furthermore, many of the same reports that recorded challenges associated with restarting NTD programmes in the context of the ongoing pandemic also noted that the need for improved planning, sensitisation, and time spent within communities actually led to improved mass drug administration coverage when programmes restarted. 6, 55, 56 This raises the question of whether now is the opportune time to critically review control strategies, particularly for high transmission settings. In the case of soil-transmitted helminths and schistosomiasis, school closures necessitated a pivot from schools-based to community-based mass drug administration, which in some settings might have improved coverage, especially for children who do not attend school. In the case of gambiense human African trypanosomiasis in the Democratic Republic of the Congo, travel restrictions necessitated vector control activities to be carried out by local teams rather than central or international teams as previously, and appropriate funding for capacity strengthening meant this was successful despite the challenges faced.^57^ Additionally, the diagnostic capacity for many NTD endemic countries has seen considerable investment and improvement to accommodate COVID-19 testing,^58^ and this could represent an opportunity to accelerate integrated surveillance for those NTD programmes that rely on individual case-finding (visceral leishmaniasis and gambiense human African trypanosomiasis).

The COVID-19 pandemic has accentuated the intrinsic connection between poverty and disease. The consequences of any delays to achieving targets, in terms of morbidity and funding (with the increased costs of restarting and also the risk of donor fatigue if programmes are not seen to be making progress)^9^ are likely to be most severe in the areas of highest endemicity. The relative effect of the timing on programmatic disruptions (ie, whether the disruption occurred near the beginning or end of a planned intervention) is predicted to vary by disease and context, which is discussed in detail elsewhere.^27^, ^29^, ^33^, ^37^ However, the overarching messages of our results are the need for programmes to come back quickly and decisively with high coverage levels, prioritising promptly delivered mitigation strategies in areas with highest infection and disease burden, and, in some cases, extending enhanced protocols beyond the initial post-COVID-19 mitigation phase should be considered.

Our results will continue to be of relevance when it comes to decision making and mitigating the effect of any future interruptions to NTD programmes, whether that be due to COVID-19, outbreaks of other diseases such as Ebola virus, or future pandemics. NTDs have a long history of a lack of prioritisation, and it is crucial that they do not again become neglected when it comes to resource allocation. Although momentous efforts over the past decade have led to substantial progress, NTDs continue to present an enormous burden of disease and disability in the world's poorest communities. The COVID-19 pandemic has presented substantial obstacles on the road towards control and elimination, but through appropriate funding, planning, and advocacy, the achievements of recent years might not be lost.

## Data sharing

No new datasets were generated or analysed for this current study, and model inputs were based on published data cited where relevant.

## Declaration of interests

This work was supported by the NTD Modelling Consortium, funded by the Bill & Melinda Gates Foundation (OPP1184344). JT and M-GB report funding from the Medical Research Council (MRC) Centre for Global Infectious Disease Analysis (MR/R015600/1), jointly funded by the UK MRC and the UK Foreign, Commonwealth & Development Office (FCDO), under the MRC–FCDO Concordat agreement, which is also part of the European and Developing Countries Clinical Trials Partnership programme supported by the EU.
