# Supplementary material for: Evaluating and mitigating the potential indirect effect of COVID-19 on control programmes for seven neglected tropical diseases: a modelling study
Source: Lancet Glob Health. 2022 Oct 11;10(11):e1600–11. doi: 10.1016/S2214-109X(22)00360-6 (PMC9579354; doi:10.1016/S2214-109X(22)00360-6)
Supplement: Supplementary appendix [file mmc1.pdf]

THE LANCET
Global Health

Supplementary appendix

This appendix formed part of the original submission and has been peer reviewed. We post it as supplied by the authors.

Supplement to: Borlase A, Le Rutte EA, Castaño S, et al. Evaluating and mitigating the potential indirect effect of COVID-19 on control programmes for seven neglected tropical diseases: a modelling study. *Lancet Glob Health* 2022; **10:** e1600–11.

APPENDIX

Evaluating the potential indirect impact of COVID-19: a modelling study of programme interruptions for seven neglected tropical diseases.

Anna Borlase, PhD*[1], Epke A. Le Rutte, PhD *[2,3,4], Soledad Castaño, PhD[3,4,5], David J Blok, PhD[2], Jaspreet Toor, PhD[1,6], NTD Modelling Consortium, Federica Giardina, PhD[2,7] and Emma L Davis, PhD[§,1,8].

* Joint first authors

§ Corresponding author: Dr. Emma Davis, Emma.L.Davis@warwick.ac.uk

[1]Big Data Institute, Li Ka Shing Centre for Health Information and Discovery, University of Oxford, Oxford, United Kingdom

[2] Department of Public Health, Erasmus MC, University Medical Center Rotterdam, Rotterdam, The Netherlands

[3] Department of Epidemiology and Public Health, Swiss Tropical and Public Health Institute, Basel, Switzerland

[4] University of Basel, Basel, Switzerland

[5] LYO-X GmbH, Allschwil, Switzerland

[6] MRC Centre for Global Infectious Disease Analysis, Department of Infectious Disease Epidemiology, School of Public Health, Imperial College London, London, UK

[7] Radboud University Medical Center, Department of Health Evidence, Netherlands

[8] Mathematics Institute, University of Warwick, Coventry, UK

Group author list: Maryan Aliee[1], Roy M Anderson[2], Diepreye Ayabina[2,3], Maria-Gloria Basáñez[2], Seth Blumberg[4], Rocio M Caja Rivera[5,6], Nakul Chitnis[7,8], Luc E Coffeng[9], Christopher N Davis[1], Michael Deiner[4], Peter J Diggle[11], Claudio Fronterre[11], Emanuele Giorgi[11], Matthew Graham[12,13], Jonathan ID Hamley[2], T Deirdre Hollingsworth[13], Matt J Keeling[1], Klodeta Kura[2], Thomas Lietman[4], Veronica Malizia[9,10], Graham F Medley[12], Edwin Michael[5,6], S Mwangi Thumbi[14,15,16], Nyamai Mutono[14,16], Travis Porco[4], Joaquín M Prada[17], Kat S Rock[1], Swarnali Sharma[5,6], Simon Spencer[1], Wilma A Stolk[5], Panayiota Touloupou[18], Andreia Vasconcelos[13], Carolin Vegvari[2], Sake J de Vlas[9]

[1] Zeeman Institute for Systems Biology and Infectious Disease Epidemiology Research, University of Warwick, UK

[2] Department of Infectious Disease Epidemiology, Imperial College London, UK

[3] Centers for Disease Control and Prevention, USA

[4] Francis I Proctor Foundation, University of California, San Francisco, USA

[5] Department of Biological Sciences, University of Notre Dame, USA

[6] University of South Florida, USA

[7] Department of Epidemiology and Public Health, Swiss Tropical and Public Health Institute, Allschwil, Switzerland

[8] University of Basel, Switzerland

[9] Department of Public Health, Erasmus MC, University Medical Center Rotterdam, Rotterdam, The Netherlands

[10] Radboud University Medical Center, Department of Health Evidence, Netherlands

[11] Centre for Health Informatics, Computing and Statistics, Lancaster University, UK

[12] Centre for Mathematical Modelling of Infectious Diseases, London School of Hygiene and Tropical Medicine, UK

[13] Big Data Institute, University of Oxford, UK

[14] Center for Epidemiological Modelling and Analysis, University of Nairobi, Kenya

[15] Institute of Immunology and Infection Research, University of Edinburgh, UK

[16] Paul G Allen School for Global Health, Washington State University, US

[17] School of Veterinary Medicine, Faculty of Health and Medical Sciences, University of Surrey

[18] School of Mathematics, University of Birmingham, UK

Table of contents

TABLE OF CONTENTS **2**

A1. PRIME-NTD TABLE **4**

A2. BOUNCE-BACK RATES FOR EACH DISEASE: ADDITIONAL RESULTS **4**

A3. SOIL-TRANSMITTED HELMINTHS: ADDITIONAL MODEL DETAILS AND SUPPLEMENTARY RESULTS **5**

A3.1 MODELS 6
A3.1.1 ERASMUS MC MODEL 6
A3.1.2 IMPERIAL COLLEGE LONDON MODEL 6
A3.2 MODEL ASSUMPTIONS AND PARAMETRIZATION 6
A3.2.1 Density dependent fecundity *6*
A3.2.2 Exposure and Contribution *6*
A3.2.2 Model Parameters *7*

A4. SCHISTOSOMIASIS (*SCHISTOSOMA MANSONI*): ADDITIONAL MODEL DETAILS AND SUPPLEMENTARY RESULTS **10**

A4.1 GOAL 10
A4.2 METHOD 10

A5. LYMPHATIC FILARIASIS: ADDITIONAL MODEL DETAILS AND SUPPLEMENTARY RESULTS **11**

A5.1. LYMPHATIC FILARIASIS MODEL DETAILS AND PARAMETERS 11
A5.1.1 TRANSFIL model description and methods *11*
A5.1.2 LYMFASIM mode description and methods *11*
A5.1.3 EPIFIL model description and methods *11*
A5.2 ALTERNATIVE SCENARIOS 12

A6. ONCHOCERCIASIS: ADDITIONAL MODEL DETAILS AND SUPPLEMENTARY RESULTS **14**

A6.1 ONCHOSIM MODEL DESCRIPTION AND PARAMETERS 14
A6.2 EPIONCHO-IBM MODEL DESCRIPTION AND PARAMETERS 16
A6.3 MODELLING APPROACH, SCENARIOS AND MITIGATION STRATEGY 18
A6.3.1 Pre-control mf prevalence setting *18*
A6.3.2 Scenarios *18*

A7. TRACHOMA: ADDITIONAL MODEL DETAILS AND SUPPLEMENTARY RESULTS **19**

A7.1 TRACHOMA MODEL DETAILS AND PARAMETERS 19
A7.2. MODELLING APPROACH, SCENARIOS AND MITIGATION STRATEGIES. 21
A7.3 ADDITIONAL RESULTS 21

A8. VISCERAL LEISHMANIASIS: ADDITIONAL MODEL DETAILS AND SUPPLEMENTARY RESULTS **22**

A8.1 MODEL STRUCTURE 22
A8.2 MODEL DESCRIPTION 23
A8.3 MODEL FITTING 23
A8.4 BOUNCE-BACK 23
A8.5 DELAYS TO THE TARGET 24
A8.6 TIMELINES OF DELAY TO THE ELIMINATION TARGET 24

A9. GHAT: ADDITIONAL MODEL DETAILS AND SUPPLEMENTARY RESULTS **25**

A9.1 MODELLING APPROACH, SETTINGS AND INTERRUPTION SCENARIOS SIMULATED 25
A9.2 ADDITIONAL RESULTS 26
A9.2.1. YEARS TO ELIMINATION OF TRANSMISSION 26
A9.2.2. NEW INFECTIONS UNDER DIFFERENT SCENARIOS 26
A9.3 MODEL S 27
A9.3.2 Parameter values *28*
A9.3.2 Summary of previous fitting *29*
A9.4 MODEL W 30

A9.4.1 Description *30*
A9.4.2 Parameter values *32*
A9.4.3 Summary of previous fitting *32*

A10 REFERENCES........ 34

A1. PRIME-NTD table

Table A1.1. PRIME-NTD (Policy-Relevant Items for Reporting Models in Epidemiology of Neglected Tropical Diseases) Summary Table

Principle	What has been done to satisfy the principle?	Where in the manuscript is this described?
1. Stakeholder engagement	The work has been discussed at the following webinars:<br>1. Neglected Tropical Diseases and COVID-19: Impact on Programme Implementation (WHO) May 2020.<br>2. A Research Agenda for NTD Programmes Affected by the COVID-19 Pandemic (WHO) June 2020.<br>3. Modelling the Impact of COVID-19 Interruptions on NTD Programmes (RSTMH) Sep 2021.<br>The following stakeholders were also engaged throughout the study design and analysis:<br>1. Control of Neglected Tropical Disease Team, WHO<br>2. Sightsavers<br>3. PATH India<br>4. The Carter Center<br>5. International Trachoma Initiative	Acknowledgements and Supplementary
2. Complete model documentation	The scenarios modelled in this study are described in the manuscript. Transmission models used are described in the manuscript and fully documented in previous publications.	Methods and References
3. Complete description of data used	No data was used explicitly. Parameters used are described in the manuscript and supplementary information. Where epidemiological scenarios were motivated by particular examples, references have been provided in the Main Text to document this motivation.	Methods and Supplementary
4. Communicating uncertainty	We account for structural uncertainty by comparing predictions across multiple models for each disease that differ in their structural assumptions regarding regulation of infection processes in humans, age- and sex- specific exposure patterns, and other factors (Figure 1). Wider consideration of stochastic uncertainty is discussed in the Supplementary Methods and Results.	Results and Supplementary
5. Testable model outcomes	The temporal infection trends and age profiles for infection predicted by the models have the potential to be tested empirically.<br>For SCH the model outcomes can be tested by the ongoing Geshiyaro project.<br>For VL the model predictions can be compared to KAMIS data in the future, with careful consideration of discrepancies between real and detected incidence.	Results and Discussion

A2. Bounce-back rates for each disease: Additional results

To enable broad comparison of the resurgence dynamics, a single year of interventions (MDA and/or active case detection and vector control) was simulated and the “bounce back” (compared to baseline) over a 10-year period

was plotted for each disease (Figure A2.1). This is shown as the mean prevalence/incidence as a fraction of baseline prevalence/incidence.

Prevalence for five out of the nine (if the soil-transmitted helminths are considered separately) diseases returns to baseline levels within 2 years of a single intervention (ascaris, hookworm, trichuris, schistosomiasis and onchocerciasis). In comparison, LF and gHAT have both not returned to their baseline endemicity levels within 5 years. These slower bounce-back rates are reflected by these diseases having the lowest predicted delay to reaching control targets following the COVID-19 interruption.

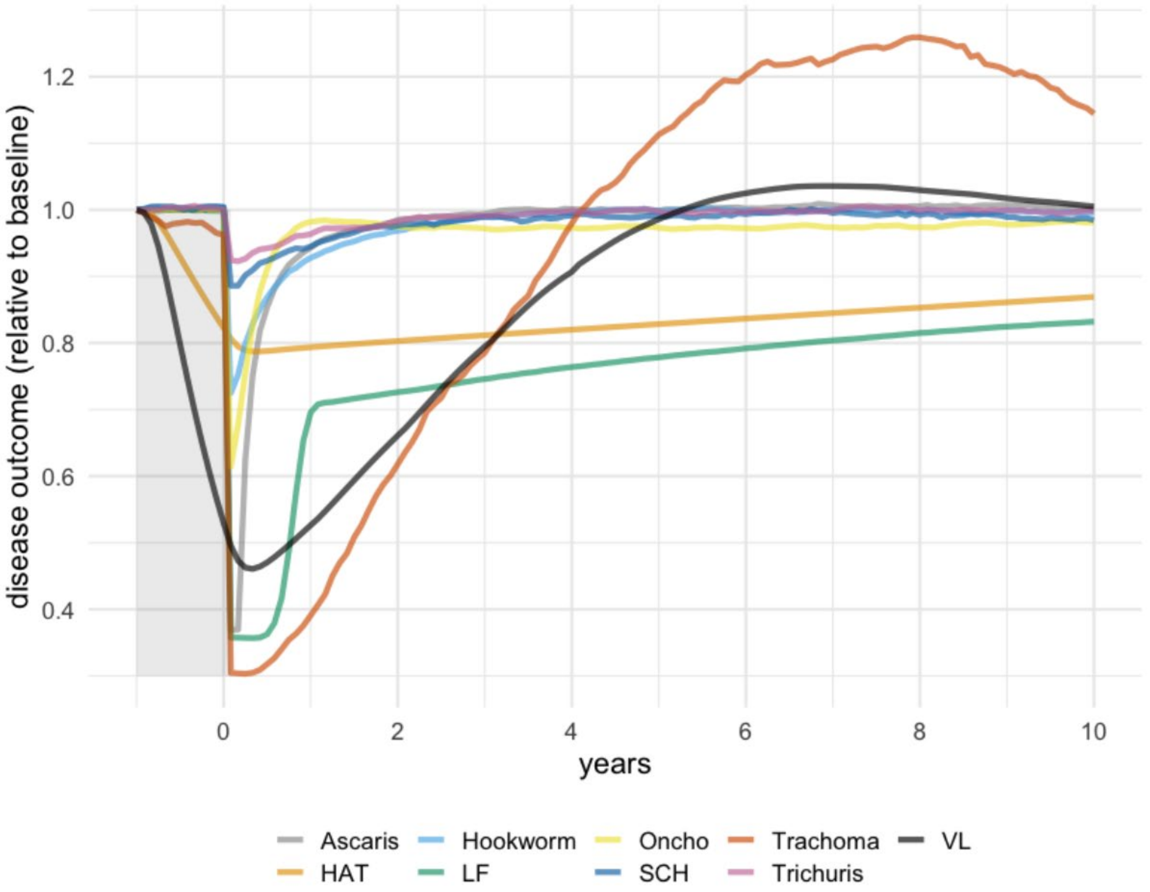

Figure A2.1. Bounce-back trajectories (starting at year 0) following one year of intervention at baseline. Disease outcomes are: mf prevalence (LF and oncho); prevalence in SAC (STH and *S. mansoni*); infection prevalence in children aged 1-9 (trachoma); population-scaled annual incidence (gHAT and VL).

A3. Soil-transmitted helminths: Additional model details and supplementary results

Table A3.1. Timeline (years) to 2030 target for hookworm and Ascaris for no interruption; a 6-month, 12-month and 18-month interruption.

	Years to target: No interruption	Years to target: 6-m. interruption	Years to target: 12-m. interruption	Years to target: 18-m. interruption
**Hookworm**				
Erasmus MC<br>Imperial CL	8·1<br>0·4	8·5<br>0·7	8·3<br>1·6	8·6<br>1·8
**Ascaris**				
Erasmus MC<br>Imperial CL	6·4<br>8·0	6·9<br>8·6	7·7<br>9·9	7·8<br>10·4

A3.1 Models

The two STH transmission models used for this work were developed independently by research groups at Imperial College London (ICL) and Erasmus MC, University Medical Center Rotterdam (Erasmus MC). Both models are stochastic individual-based models (IBMs) and are based on similar biological and demographic assumptions.[1,2] Model output at a given time can therefore be at individual level (worm burden, egg output) or at population level or for individuals of a particular age (mean worm burden, prevalence of infection). Model output can be expressed as probability distributions and can be directly compared to observed epidemiological data, for the purposes of parameter estimation and model validation, as well as for model comparisons.

A3.1.1 Erasmus MC model

The Erasmus MC model (WORMSIM) is stochastic and individual-based, in terms of both hosts and intestinal parasite numbers per host. WORMSIM simulates the life histories of a discrete number of individual humans and individual worms within those humans, which are born and die in a stochastic fashion. Simulated humans are exposed and contribute to a central reservoir of infection in the external environment, in which infective material (e.g. worm larvae or eggs) survive in an exponential fashion (at each time step in the simulation, a fixed proportion of the reservoir decays). Infective material is produced by female worms after a period of pre-patency (maturation in the human host), and only when at least one male worm is present in the same host. The degree of parasite aggregation within the human population is governed by the level of inter-individual variation in exposure to the central reservoir of infection (by age, sex, and random individual factors). Similarly, the model allows for heterogeneity in participation in PC, as well as systematic non-compliance to PC. The model further accounts for different sources of variation, such as measurement error in parasitological test outcomes (any arbitrary parasitological test based on egg counts can be simulated, e.g. Kato-Katz faecal smear. Model code and installation and user instructions have been published elsewhere.[3]

A3.1.2 Imperial College London model

The model simulates the number of worms present in each individual person in a village over time. Individuals contribute to and can acquire infections from the environmental reservoir of infective stages (eggs or larvae). The transmission parameters are age- and species-dependent. The number of worms in an individual follows a negative binomial distribution, i.e. a large proportion of the population have a few worms and a small proportion of the population have many worms. Further details of the model can be found in.[4]

A3.2 Model assumptions and parametrization

A3.2.1 Density dependent fecundity

At high host worm burdens, egg production per worm is restricted by overcrowding effects (density dependent fecundity), as recorded in field epidemiological studies involving worm expulsion and faecal egg sampling. In the EMC description, egg production gradually levels off to a maximum level with increasing worm burden. In the ICL description, overcrowding effects lead to the maximum egg production rate being achieved earlier and a subsequent small drop in production for higher worm burdens.

A3.2.2 Exposure and Contribution

Different assumptions about the relative contribution of different age groups to transmission (i.e. the relative frequency of practicing open defaecation; the models employ very similar assumptions about exposure to the environmental reservoir, leading to nearly identical predictions for age profiles in infection level). Based on the age pattern in hookworm infection levels, the EMC model assumes that the practice of defaecation increases with age up to age ten, and this pattern in open defaecation is then also applied to *Trichuris* and *Ascaris*. In contrast, the ICL model assumes that age-dependent contribution is proportional to age-dependent exposure (i.e. and therefore differs between the three worm species). As such, given identical infection levels in by age, in the EMC model adults contribute relatively more to transmission than in the ICL model, reducing the effectiveness of SAC-targeted MDA, but making community-wide treatment more beneficial.[5]

A3.2.2 Model Parameters

Table A3.2. Model parameters used to simulate transmission of Ascaris lumbricoides, Trichuris trichiura and hookworm infections.

	**Value or assumption**	
**Parameter**	**Erasmus MC**	**Imperial College London**
**Human demography**		
Hookworm	Demographic data quantified for sub-Saharan Africa 2000 United Nations Population Division[6]	Demographic data taken from 2003 Kenya Demographic and Health Surveys.
Ascariasis	Indian fertility and mortality rates as reported for 1980-1985 by United Nations Population Division (2015 Revision).	Demographic data taken from 2003 Kenya Demographic and Health Surveys.
Trichuris	Indian fertility and mortality rates as reported for 1980-1985 by United Nations Population Division (2015 Revision).	Demographic data taken from 2003 Kenya Demographic and Health Surveys.
**Transmission of infection**		
Seasonal variation in contribution to reservoir	Stable throughout the year (assumption).	Stable throughout the year (assumption).
**Aggregation of parasites in hosts**		
Hookworm	$k_w = 0 \cdot 35$.[7]	$k_w = 0 \cdot 35$.[7]
Ascariasis	$k_w = 0 \cdot 8$.[8]	$k_w = 0 \cdot 8$.[4,8]
Trichuris	$k_w = 0 \cdot 38$ in high prevalence settings, fitted to data from[9], $k_w = 0 \cdot 12$ in moderate prevalence settings.	$k_w = 0 \cdot 38$ in high prevalence settings, fitted to data from[9], $k_w = 0 \cdot 12$ in moderate prevalence settings.
**Variation in exposure and contribution to the environmental reservoir by age and sex**		
Hookworm	Relative exposure and contribution to the reservoir both increase linearly from 0 to 1 between ages 0–10 and is stable thereafter with no difference between males and females[3].	Relative exposure and contribution to the reservoir are assumed to vary be constant across age groups, assuming no difference between males and females. The values were estimated from baseline data of the Tumikia study[10] and unpublished epidemiological data of the DeWorm3 study.[11]
Ascariasis	Contribution to the reservoir increases linearly from 0 to 1 between ages 0–10 and is stable thereafter with no difference between males and females (reflecting behaviour related to defaecation and mobility patterns as previously estimated for hookworm[3]). Exposure to the reservoir is defined as a piece-wise linear function of age that increases linearly from a base level x_0 =0·33 of relative exposure at age zero to a relative exposure of 1·0 at age a_{peak}=3, and then again linearly declines back to the base level x_0 at age 15 and is stable thereafter. This function aims to reflect behaviour leading to ingestion of contaminated matter, which typically peaks in young children.[8]	Relative exposure and contribution to the reservoir by age are assumed to be equal and are estimated from the baseline data: 0·22 (0-4 years), 1·88 (5-9), 1·0 (10-19), 0·53 (20+).
Trichuris	Contribution to the reservoir increases linearly from 0 to 1 between ages 0–10 and is stable thereafter with no difference between males and females (reflecting behaviour related to defaecation and mobility patterns as previously estimated for hookworm[3]). Exposure to the reservoir is defined as a piece-wise linear function of age that increases linearly from a base level x_0 =0·33 of relative exposure at age zero to a relative exposure of 1·0 at age a_{peak}=3, and then again linearly declines back to the base level x_0 at age 15 and is stable thereafter. This function aims to reflect behaviour leading to ingestion of contaminated matter, which typically peaks in young children.[8]	Relative exposure and contribution to the reservoir are assumed to vary piece-wise constant by age group and are estimated at 0·3 (0-4 years), 1·28 (5-14), 1 (15-24) and 0·17 (ages 25+), assuming no difference between males and females. These figures were estimated from epidemiological data from [9].
**Life history and productivity of the parasite in the human host**		
**Average worm lifespan**		
Hookworm	3 years[12–14]	2 years[2]
Ascariasis	1 year[8,12–15]	1 year[12–15]

Trichuris	1 year[2,9]	1 year[2,9]
Variation in worm lifespan	Weibull distribution with shape 2; i.e. the mortality rate is zero at age zero and then increases linearly with worm age (assumption as previously used for hookworm[3]).	Exponential distribution: i.e. the mortality rate is constant and independent of worm age.
Pre-patent period		
Hookworm	7 weeks[12,13,16,17]	No pre-patent period used.
Ascariasis	10 weeks[12]	No pre-patent period used.
Trichuris	10 weeks[12]	No pre-patent period used.
Age-dependent reproductive capacity	Constant over age (assumption).	Constant over age (assumption).
Female worm fecundity	Density-dependent on total number of female worms in host, assuming hyperbolic saturation[3].	Density-dependent on total number of female worms in host, assuming exponential saturation. Exponential model of saturation with parameter $\gamma = 0{\cdot}02$ for hookworm[18], $\gamma = 0{\cdot}07$ for ascaris[4], and $\gamma = 0{\cdot}0035$ for trichuris[9,19].
Hookworm	On average 8·3 eggs per female worm per 41·7 mg sample of faeces (200 epg per female worm, as previously reported based on association between number of expulsed adult female worms and egg counts based on Kato-Katz). The average maximum total host output is assumed to be 62·5 eggs per 41·7 mg faeces (1500 epg, as previously assumed[3]).	On average 3 eggs per female worm per 41.7 mg sample of faeces (72 epg per female worm, as previously reported based on association between number of expulsed adult female worms and egg counts based on Kato-Katz[20]).
Ascariasis	On average 406 eggs per female worm per 41·7 mg sample of faeces (9750 epg per female worm), and maximum total host output of 777 eggs per 41·7 mg faeces on average (18,650 epg). These figures were estimated from pre-control data on number of expulsed adult female worms and egg counts based on a concentration and sedimentation technique using homogenised stools[8].	On average 320 eggs per female worm per 41·7 mg sample of faeces (7674 epg per female worm).
Trichuris	On average 15·4 eggs per female worm per 41·7 mg sample of faeces (370 epg per female worm), and maximum total host output of 3333·33 eggs per 41·7 mg faeces on average (80,000 epg). These figures were estimated from pre-control data on number of expulsed adult female worms and egg counts based on a concentration and sedimentation technique using homogenised stools[8].	On average 5·875 eggs per female worm per 41·7 mg sample of faeces (141 epg per female worm)[3].
Host immunity to incoming infections	None (assumption).	None (assumption).
**Infection dynamics in environmental reservoir**		
Survival of infective material in the central reservoir	Exponential survival (assumption).	Exponential survival (assumption).
Hookworm	Average lifespan of two weeks, implemented as a monthly survival probability of $\exp(-26/12) = 11 \cdot 5\%$ (95%-CI: 0·05–7·38 weeks under assumption of exponential survival), based on the notion that average survival time is in the order of weeks.[16,17,21]	Average lifespan of 30 days.[2]
Ascariasis	Average lifespan of 1·5 month, implemented as a monthly survival probability of $\exp(-1/1 \cdot 5) = 51 \cdot 3\%$ (95%-CI: 0·04–5·53 months under assumption of exponential survival).[13,14]	Lifespan of approximately 2 months.[1]
Trichuris	Average lifespan of 20 days implemented as a monthly survival probability of $\exp(-1/(2/3)) = 22 \cdot 3\%$ (95%-CI: 0·02–2·46 months under assumption of exponential survival.	Lifespan of approximately 20 days.[9]
**Drug treatment**		
Proportion of adult worms killed by single dose of albendazole (400 mg), or pyrantel pamoate (10 mg/kg, ascariasis only)	Assumption: proportion killed is equal to the faecal egg reduction rate.	Assumption: proportion killed is equal to the faecal egg reduction rate.

Hookworm	0·95 for albendazole[22]	0·95 for albendazole[22]
Ascariasis	0·99 for albendazole[22]	0·99 for albendazole[22]
Trichuris	0·60 for albendazole[22]	0·60 for albendazole[22]
**Diagnostic test outcomes**		
**Variability in measured host load of infective material (eggs per examined sample of faeces)**		
Hookworm	Kato-Katz: negative binomial distribution with aggregation parameter $k = 0 \cdot 35$, estimated separately from repeated individual-level egg count data from Uganda.[23]	Kato-Katz: negative binomial distribution with aggregation parameter $k = 0 \cdot 35$, estimated from unpublished triple egg count data from Tamil Nadu, India
Ascariasis	Kato-Katz: negative binomial distribution with aggregation parameter $k = 0 \cdot 25$.	Kato-Katz: negative binomial distribution with aggregation parameter $k = 0 \cdot 3$ [24]
Trichuris	Kato-Katz: negative binomial distribution with aggregation parameter $k = 0 \cdot 82$.	Kato-Katz: negative binomial distribution with aggregation parameter $k = 0 \cdot 82$ [19]
**Cut-offs for no, light, moderate, and heavy infection**		
Hookworm	1, 2000, and 4000 epg	1, 2000, and 4000 epg
Ascariasis	1, 5000, and 50,000 epg	1, 5000, and 50,000 epg
Trichuris	1, 1000, and 10,000 epg	1, 1000, and 10,000 epg

A4. Schistosomiasis (*Schistosoma mansoni*): Additional model details and supplementary results

A4.1 Goal

The 2030 goal for schistosomiasis is elimination as a public health problem (EPHP), achieved when the prevalence of heavy-intensity (eggs per gram, epg ≥ 400 for *Schistosoma mansoni*) infections in school-age children (SAC; 5-14 year olds) is reduced to less than 1%.[25]

A4.2 Method

We used an age-structured deterministic model developed by Imperial College London.[26] The model incorporates treatment by mass drug administration (MDA) with praziquantel and is parameterised for *S. mansoni* using previously published parameter values (Table A4.1).[27]

We considered a moderate (30% baseline prevalence among SAC) and a high (70% baseline prevalence among SAC) prevalence setting for *S. mansoni*. We used two different age intensity profiles with low and high adult burdens of infection relative to SAC (Figure A4.1). In the model, we implemented annual MDA to 75% SAC only. We assumed no non-adherence and no non-access to treatment, i.e. MDA was delivered at random at each round of treatment. We also assumed no migration, i.e. single community with a population size of 1000. Simulations were carried out for missing the second round of MDA (compared to not missing MDA) and used to determine the time taken to achieve EPHP.

For each transmission setting and age profile, the simulations were run for 15 years. For each point in time, we determined the prevalence of heavy-intensity infections in SAC to investigate whether EPHP had been achieved.

Note that although *S. haematobium* was not modelled in this investigation, as this species typically has a low adult burden of infection (Figure A4.1), the results are likely to be similar to *S. mansoni* with a low adult burden of infection.

SCHISTOX, an individual-based stochastic model for the study of schistosome transmission dynamics and the impact of control by mass drug administration has been developed by the University of Oxford and is publicly available for use (https://github.com/mattg3004/Schistoxpkg.jl, https://github.com/mattg3004/SchistoIndividual).[28] This was used to produce the bounce-back Figure A2.1.

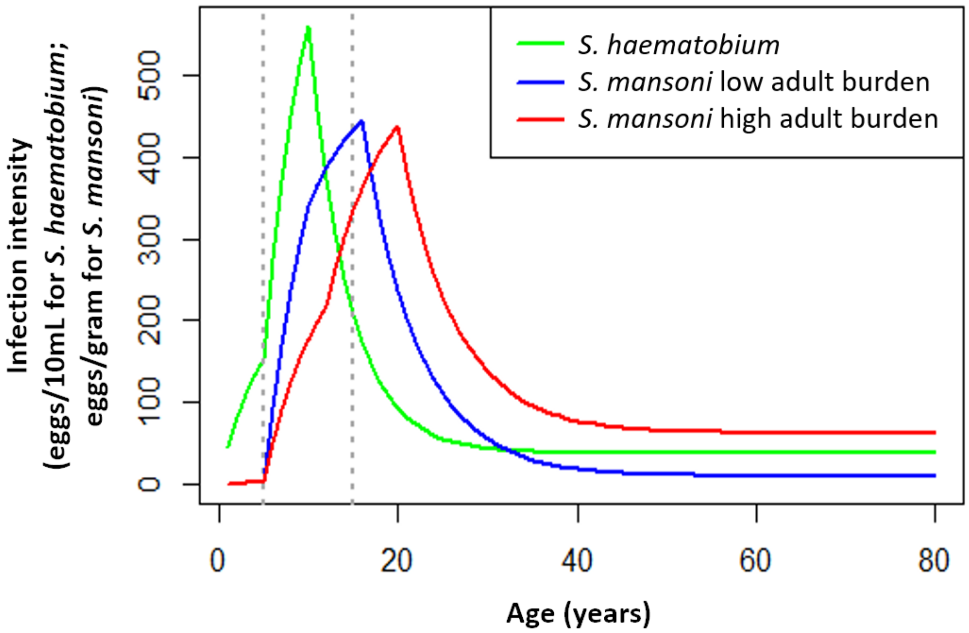

Figure A4.1. Age-intensity profiles of infection for *Schistosoma mansoni* using model-simulated low and high adult burdens of infection (relative to school-aged children [SAC; 5–14 years old]) and *S. haematobium* using previous fit to data.[29] Figure adapted from [30]; http://creativecommons.org/licenses/by/4.0/.

Table A4.1. Parameter values used for *Schistosoma mansoni*.

Parameter	Value	Source
Fecundity	0·34 eggs/female/sample	[1,31,32]
Egg distribution within the individual	0·87	[31,32]
Aggregation parameter	0·24	[26,33]
Density dependent fecundity	0·0007/female worm	[26,34]
Worm lifespan	5·7 years	[1,35]
Drug efficacy	86·3%	[36]
Low adult burden setting: Age specific contact rates for 0-4, 5-9, 10-15, 16+ years old	0·01, 1·20, 1, 0·02	[34]
High adult burden setting: Age specific contact rates for 0-4, 5-11, 12-19, 20+ years old	0·01, 0·61, 1·00, 0·12	[34]
Prevalence of infection	Percentage of population having > 0 eggs per gram [epg]	-
Prevalence of heavy-intensity infections	Percentage of population having ≥ 400 epg	[25,37]
Human demography	Based on Uganda's demographic profile	[13,23]

A5. Lymphatic filariasis: Additional model details and supplementary results

A5.1. Lymphatic filariasis model details and parameters

Full model descriptions for all three models, including parameters, are given in the supplementary information of Prada *et al.* (2019).[38]

A5.1.1 TRANSFIL model description and methods

The mathematical model of lymphatic filariasis (LF) transmission TRANSFIL is a stochastic individual-based model of LF infection in human populations, simulating worm burden, microfilaraemia and other demographic parameters relating to age and risk of exposure. Humans are modelled individually, with their own male and female worm burden. The concentration of mf in the peripheral blood is modelled for each individual and increases according to the number of fertile female worms as well as decreasing at a constant rate. The total mf density in the population contributes towards the current density of L3 larvae in the human-biting mosquito population, where the distribution of L3 amongst the human-biting mosquito population is completely homogeneous.

An empirically derived relationship is used for the uptake of mf by a mosquito, where both *Culex* and *Anopheles* uptake curves are implemented depending on setting (see Irvine *et al.*).[39] The model dynamics are therefore divided into the individual human dynamics, including age and turnover; worm dynamics inside the host; microfilariae dynamics inside the host and larvae dynamics inside the mosquito.

A5.1.2 LYMFASIM mode description and methods

LYMFASIM[40,41] is a stochastic individual-based model for lymphatic filariasis (LF). It is a specific model variant within WORMSIM, a generalized framework for modelling transmission and control of helminth infections in humans.[3,42] LYMFASIM simulates the life histories of individual people and individual worms in a community, and the effects of interventions (e.g. mass drug administration, integrated vector management, bednet use) on transmission and morbidity, while taking into account the human demography and the complexities of helminth transmission. The model has been described elsewhere and has been applied to support decision making on control and elimination of lymphatic filariasis in different settings.[19,20,23–31]

Mass drug administration (MDA) is simulated by specifying the exact timing of the treatment rounds (year, month), the efficacy of the applied treatment regimen, the achieved coverage level, and compliance patterns. LYMFASIM assumes that a fraction of people never participates in MDA (e.g. systematic refusal, related to chronic illness). In addition, LYMFASIM allows the relative compliance to vary between age and sex groups; this mechanism captures transient contra-indications for MDA (e.g. exclusion of young children and pregnant women) and other age- and sex-related behavioral factors driving participation in MDA. Lastly, each individual has a personal inclination to participate in MDA, which is considered as a lifelong property. A stochastic process eventually defines for each individual whether they are treated in a given round, depending on the calculated probability.

A5.1.3 EPIFIL model description and methods

EPIFIL is a hybrid coupled partial differential and differential equation model that tracks changes in the pre-patent and adult worm burdens and microfilariae levels in the human host, as well as the average number of infective L3 larval stages per mosquito. The model also includes a measure of immunity developed by human hosts against L3 larvae. The model has been previous well described and parameterised.[52–58]

Intervention by mass drug administration was modeled based on the assumptions that anti-filarial treatment with a combination drug regimen acts by killing certain fractions of the populations of adult worms and microfilariae instantly after the drug administration.

A5.2 Alternative scenarios

Table A5.1. Timeline (years) to 2030 target across all three models (LYMFASIM, EPIFIL and TRANSFIL). Mitigation strategy is one additional round of MDA after restarting the programme.

**Endemicity setting**<br>**% mf baseline prevalence**	**Years to target:**<br>**No interruption**	**Years to target:**<br>**12-month interruption, no mitigation**	**Years to target:**<br>**12-month interruption, with mitigation**
**LYMFASIM, Erasmus MC - *W. bancrofti*, IA settings (*Anopheles*)**			
High: 15-20% (95% CIs)	13·0 (7-26)	13·7 (0-37)	13·2 (0-37)
Med: 5-10% (95% CIs)	8·4 (2-25)	9·2 (0-40)	8·3 (0-40)
**LYMFASIM, Erasmus MC - W. bancrofti, DA settings (*Culex*)**			
High: 15-20% (95% CIs)	9·1 (5-25)	9·7 (0-37)	9·2 (0-39)
Med: 5-10% (95% CIs)	5·3 (2-23)	6·2 (0-35)	5·3 (0-35)
**EPIFIL, Notre Dame - *W. bancrofti*, IA settings (*Anopheles*)**			
High: 15-20%	7·7 (7-9)	8·7 (8-10)	7·8 (7-9)
Med: 5-10%	5·4 (4-6)	6·4 (5-7)	5·0 (4-7)
**EPIFIL, Notre Dame - *W. bancrofti*, DA settings (*Culex*)**			
High: 15-20%	6·7 (6-7)	7·7 (7-9)	6·7 (6-8)
Medium: 5-10%	4·9 (4-6)	5·9 (5-7)	4·3 (4-6)
**TRANSFIL, Oxford/Surrey/Warwick - *W. bancrofti*, IA settings (*Anopheles*)**			
High (15-20%)	11·4 (7-23)	12·0 (8-23)	11·5 (7-24)
Medium (5-10%)	7·7 (5-13)	8·4 (6-14)	7·6 (4-13)
**TRANSFIL, Oxford/Surrey/Warwick - *W. bancrofti*, DA settings (*Culex*)**			
High: 15-20% (95% CIs)	10·9 (6-23)	11·8 (7-23)	11·0 (6-22)
Med: 5-10% (95% CIs)	7·2 (4-18)	8·0 (5-19)	7·2 (4-18)

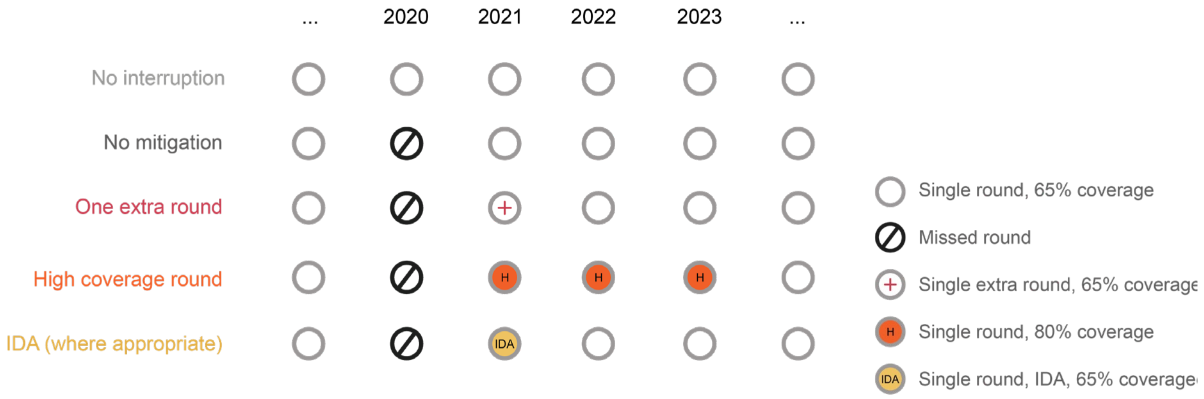

Figure A5.1. Mitigation methods after missing one year of MDA. Dark grey: resume at 65% coverage. Red: one year of bMDA after the program restarts. Orange: three years increased coverage (80%) then resume 65%. Yellow: resume the program with one round IDA, then return to the previous regime (in areas using DA only).

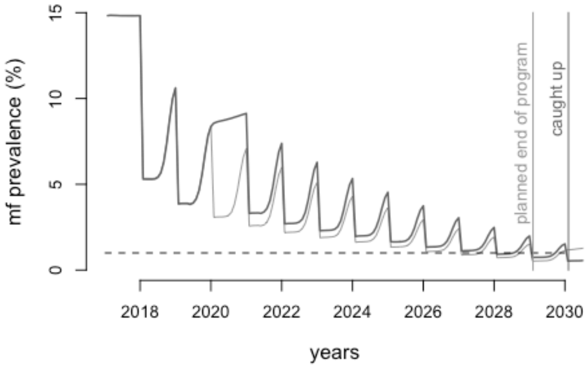
mf prevalence (%)
years
planned end of program
caught up

H

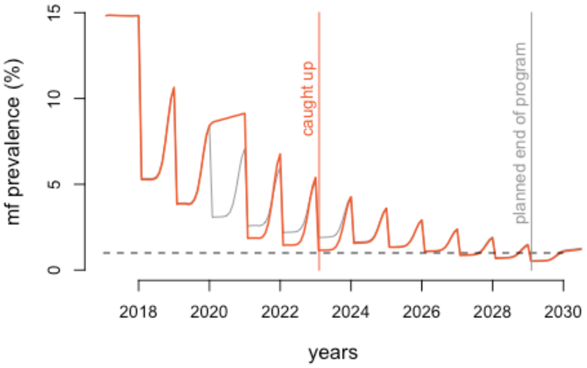
mf prevalence (%)
years
caught up
planned end of program

+

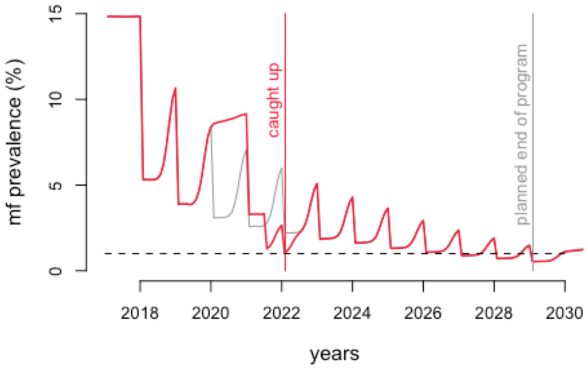
mf prevalence (%)
years
caught up
planned end of program

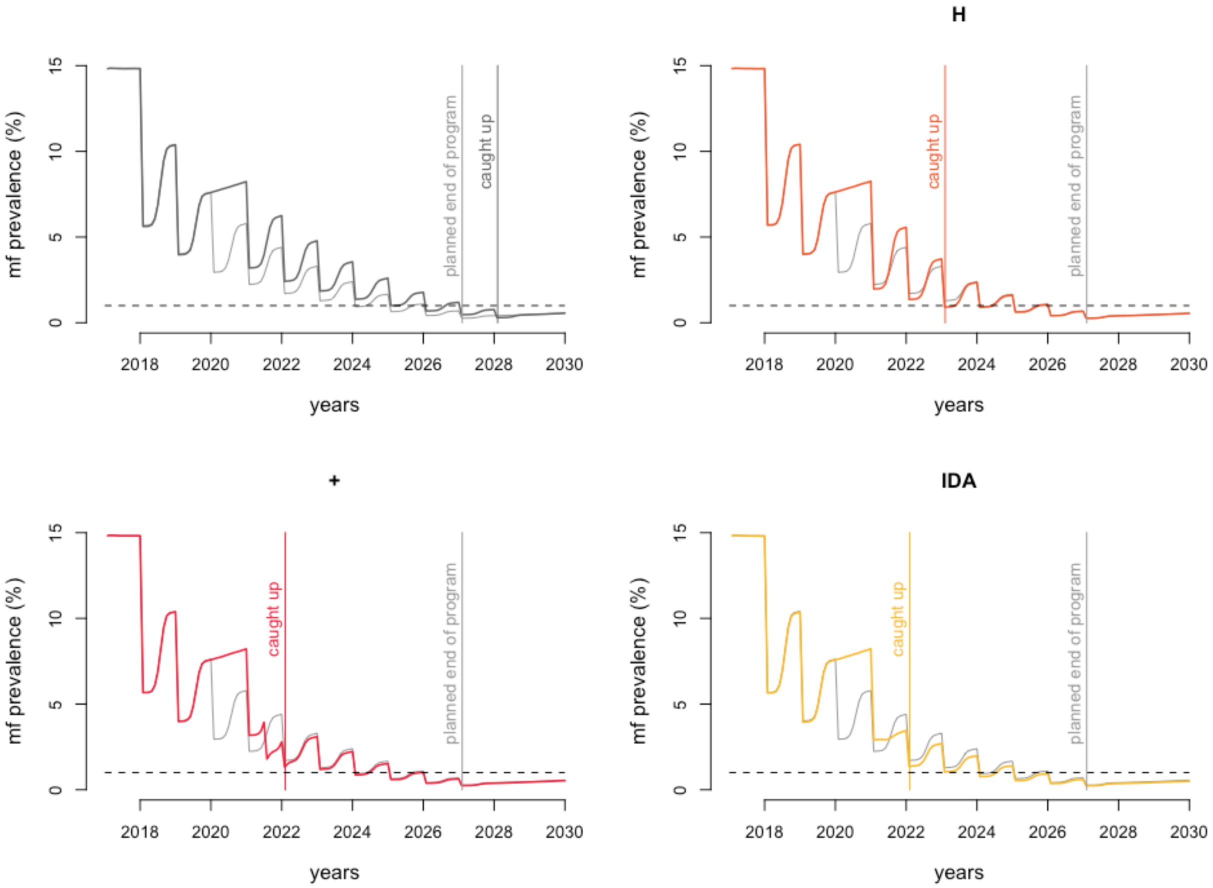

Figure A5.3. Example timelines for 15% mf prevalence (2018) with DA. Assuming one round of missed MDA in 2020 and using mitigation methods from Figure A5.1 (continue at 65%, dark grey; 3 rounds 80%, orange; 1 round biannual MDA in year 5, red; 1 round of IDA, yellow); The dashed line shows 1% mf prevalence.

A6. Onchocerciasis: Additional model details and supplementary results

Two available onchocerciasis transmission models – ONCHOSIM and EPIONCHO-IBM - are used to simulate the effect of a 6-, 12- and 18-months interruption in annual mass drug administration (MDA) with ivermectin on the infection microfilarial (mf) prevalence trends. The models have been used before to inform the World Health Organization 2030 targets for eliminating onchocerciasis (NTD Modelling Consortium Onchocerciasis Group 2019) and to explore the impact of various strategies.[59]

A6.1 ONCHOSIM model description and parameters

ONCHOSIM is a stochastic individual-based model that simulates the transmission of onchocerciasis in a closed dynamic population of approximately 440 individuals (rural village).[60,61] The model simulates life histories of human individuals and *Onchocerca volvulus* worms and mf within individual human hosts. Transmission of infection occurs through bites of blackflies whose intensity is represented by the annual biting rate. The probability that an individual is bitten by a blackfly is assumed to depend on age (exposure to blackfly increases linearly between the ages of zero and 20), sex (males have a higher exposure), personal factors such as attractiveness to blackflies, and seasonal biting variation of blackflies. At each bite, blackflies can transmit or pick up the infection. Only a small proportion of transmitted larvae will successfully develop into adult worms. Following insemination of females by male worms, new microfilariae (mf) are produced, which can be picked up by the blackfly. These mf develop in the blackfly into the infective stage (L3), which is modelled deterministically in the vector. The model accounts for infection acquired from other villages. This is captured by the parameter called external force of infection. A more detailed description of the model can be found elsewhere.[61] Information about the quantification of biological, transmission, parasitic and treatment parameters can be found in Table A6.1.

Table A6.1. ONCHOSIM parameter quantification.

Parameter	Value	Source
**Transmission of infection**		
**General transmission parameters**		
Relative biting rate (*rbr*)	Multiplied with the reference *mbr* values, to modify the monthly and annual biting rate: varied between simulations.	
Seasonal variation in contribution to reservoir (*mbr*)	Reference *mbr* values (Jan-Dec):<br>5340, 4700, 3000, 3840, 3880, 3380,<br>5260, 6820, 6020, 6580, 7500, 5380	[62] and entomological data collated by OCP
Transmission probability (v), i.e. the probability that an infective particle in the reservoir successfully develops into a parasite life stage that is capable of infecting a human host	$v = 0{\cdot}07345$; see reference for the derivation of this value, given parameters for fly biology and development of infective L3 larvae within the fly.	[63]
Success ratio (*sr*)	$sr = 0{\cdot}0031$	[64,65]
Zoophily (z, $1 - h$)	$z = 0{\cdot}04$; $h = 0{\cdot}96$	[66] and expert opinion (OCP entomologists)
**Individual relative exposure and contribution to flies**		
Relative exposure and contribution by age and sex	Zero at birth, linearly increasing between ages 0–20 from 0 to 1·0 for men and from 0 to 0·7 for women, and then constant from the age of 20 years onwards	[65]
Variation due to personal factors (fixed through life) given age and sex (α_{Exi})	Gamma distribution with mean 1·0. Shape and rate equal varies between simulations	
**Life history and productivity of the parasite in the human host**		
Average worm lifespan (*Tl*)	10 years	[67]
Variation in worm lifespan	Weibull distribution with shape 3·8.	Assumption; [67]
Prepatent period (*pp*)	1 year	[67–69]
Age-dependent microfilaria production capacity, $R(a)$	$R(a) = 1$ for $0 \leq a < 5$<br>$R(a) = 1-((a-5)/15)$ for $5 \leq a < 20$<br>$R(a) = 0$ for $a > 20$	[67,70,71]
Longevity of microfilariae within host (*Tm*)	9 months	[65]
Mating cycle (*rc*)	3 months	[65,67,72]
Male potential (*pot*)	100 female worms.	[65]
**Density-dependent female worm reproductive capacity**		
Average contribution of an inseminated worm at peak fecundity to the skin mf-density	7·6 mf/worm	[65]
Exponential saturation of individual female worm productivity per worm present in host (λ_z)	$\lambda_z = 0$ i.e. no exponential saturation.	Assumption
**Morbidity**		
Disease threshold (*Elc*) for blindness	Weibull distribution with mean 10,000 and shape 2·0	[73]
Reduction in remaining life expectancy due to blindness (*rl*)	50%	[73] which refers to partly published data from OCP; [60,74–76]
**Infection dynamics in the vector**		
L1-uptake in the vector	Exponential saturating function with parameters $a = 1{\cdot}2$, $b = 0{\cdot}0213$, and $c = 0{\cdot}0861$.	[77] which refers to [78,79]
**Mass treatment coverage**		
**Timing and coverage (C_w)**	**Varied between scenarios**	
Relative compliance ($c_r(k, s)$) by age and sex<br><br>Age-group (k): 0-4, 5-9, 10-14, 15-19, 20-29, 30-49, 50+<br>cr(k,males): 0·00, 0·75, 0·80, 0·80, 0·70, 0·75, 0·80<br>cr(k,females): 0·00, 0·75, 0·70, 0·74, 0·65, 0·70, 0·75		Based on unpublished OCP data
**Drug treatment**		
Proportion of microfilariae cleared from host	100%	[80]
Duration of temporary reduction in female reproductive capacity (Tr_0), average	11 months	[80]
Permanent reduction in female worm reproductive capacity (d_0), average	34·9%	[80]
Proportion of adult worms killed (m_0)	0%	[80]
Relative effectiveness of treatment in a person (v)	Weibull distribution with mean 1 and shape 2	[80]
**Surveys**		
Timing	Surveys are done at yearly intervals. The simulation allows for a 200-year warming-up period before the first survey in 1998.	
Dispersal factor for worm contribution to measured density of infective material (d)	Exponential distribution with mean 1	[67]
Variability in measured host load of infective material (here: mf per skin snip)	Poisson distribution with mean $ss(t)$	[60]

A6.2 EPIONCHO-IBM model description and parameters

EPIONCHO-IBM is a stochastic individual-based model, which simulates humans in a closed population, keeping track of the number of infecting adult *O. volvulus* and mf in a human. The model accounts for age- and sex-dependent exposure of humans to blackfly bites, and the individual-level variation in exposure. The production of mf requires the presence of both male and female worms, assuming a completely polygamous mating system. Mortality rates of adult worms and mf are assumed to increase with age and parasite fecundity decreases with age. Density-dependent processes are assumed to act on three stages of the *O. volvulus* lifecycle: 1) establishment of larvae within the vector; 2) parasite-induced mortality of the vector, and 3) establishment of adult worms within the human. The dynamics of the parasite within the vector are modelled deterministically at a fly population level. The model accounts for a latent period in the development of the parasite in the vector by including L1, L2 and L3 stages. A more detailed description of the model can be found elsewhere.[81] Information about the quantification of biological, transmission, parasitic and treatment parameters can be found in Table A6.2.

Table A6.2. EPIONCHO-IBM parameter quantification (Hamley *et al.* 2019).

Parameter or variable	Definition	Value and units	Reference
**Human host demography**			
N_H	Number of human hosts in population	500	[81]
μ_H	Mortality rate of human hosts	$0{\cdot}02$ year^{-1}	[82]
a_{max}	Maximum age of human hosts	80 years	[83]
ψ'_S	Probability that a human host is of sex s	$\psi'_F = \psi'_M = 0 \cdot 5$	[83]
**Exposure to blackfly bites**			
k_E, β_E	Shape and rate parameters of the gamma distribution describing individual human host exposure to blackfly bites	Vary	[81]
$Q = E_M/E_F$	Relative male to female exposure to blackfly bites	1·20	[83]
α_F	Age-specific change in contact rate with vectors for females	-0·023 year^{-1}	[83]
α_M	Age specific change in contact rate with vectors for males	0·007 year^{-1}	[83]
q	Period (age) preceding initial increase in exposure to vector bites during childhood	0 years	[83]
**Human host infection**			
$\beta = h/g$	Per blackfly biting rate on humans, calculated as the product of the proportion of blackfly bites taken on humans (the human blood index, h) and the reciprocal of the duration of the gonotrophic cycle, g	$h = 0{\cdot}63$[§] $g = 1/104$ years	[82,84]
$ABR = \beta V/H$	Annual biting rate of blackflies on humans; the key variable for simulating different endemicity levels	Varies; bites/person/year	[81]
$ATP(t) = ABR \times L3(t)$	Annual transmission potential of blackflies to humans	Defined by ABR and $L3(t)$	[82]
$\Pi_{H(i)}\left[ATP(t-\tau_H), \Omega_T(a_{(i)}-\tau_H)\right] = \left[\frac{\delta_{H0}+\delta_{H\infty}c_H ATP(t-\tau_H)\Omega_T(a_{(i)}-\tau_H)}{1+c_H ATP(t-\tau_H)\Omega_T(a_{(i)}-\tau_H)}\right]$	Density-dependent constraint on the proportion of infective L3 larvae successfully establishing as adult worms	defined by δ_{H0}, $\delta_{H\infty}$, c_H, $ATP(t-\tau_H)$ and $\Omega_T(a_{(i)}-\tau_H)$	[82,83,85]
δ_{H0}	Proportion of L3 larvae developing to the adult stage within the human host, per bite, when $ATP(t) \to 0$	Dimensionless, varies	Re-estimated in [81]
$\delta_{H\infty}$	Proportion of L3 larvae developing to the adult stage within the human host, per bite, when $ATP(t) \to \infty$	Dimensionless, varies	Re-estimated in [81]
c_H	Severity of transmission intensity-dependent parasite establishment within humans	varies	Re-estimated in [81]
τ_H	Time delay between L3 entering the host and establishing as adult worms	0·8 years	[86]
**Parasite demography**			
y_W	Parameter relating mortality rate to age in adult worms, Eq (S6), (S7)	0·1	[81]

d_W	Parameter relating mortality rate to age in adult worms, Eq (S6), (S7)	6·01	[81]
y_M	Parameter relating mortality rate to age in microfilariae, Eq (S6), (S7)	1·09	[81]
d_M	Parameter relating mortality rate to age in microfilariae, Eq (S6), (S7)	1·43	[81]
L_W	Maximum longevity of adult worms	20 years	[67]
L_M	Maximum longevity of microfilariae	2·5 years	[87]
c_{max}	Number of discrete age classes in adult worms and microfilariae	21	[81]
q_M	Duration of each age class for microfilariae	0·125 years	[81]
q_W	Duration of each age class for adult worms, Eq (S8)	1 year	[81]
ε^*	Per capita rate of production of microfilariae per mg of skin per (fertile) adult female *Onchocerca volvulus* at age zero, Eq (S11)	1·15 year^{-1}	[88]
ω	Per capita rate of progression from non-fertile to fertile adult female *O. volvulus,* Eq (S9)	0·59 year^{-1}	[88,89]
λ_0	Per capita rate of reversion from fertile to non-fertile adult female *O. volvulus,* Eq (S10)	0·33 year^{-1}	[88,89]
F	Parameter relating parasite fecundity to age, Eq (S11)	70	[81]
G	Parameter relating parasite fecundity to age, Eq (S11)	0·72	[81]
Larval stages within the vector and adult female blackfly population dynamics.			
$\Pi_{V(i)}(t) = \frac{\delta_{V0}}{[1 + c_V M_{(i)}(t)\Omega_T(a_{(i)})]}$	Proportion of microfilariae (mf) per mg of skin in human host i developing into infective L3 larvae within the blackfly vector per bite	Defined by δ_{V0}, c_V, $M_{(i)}(t)$ and $\Omega_T(a_{(i)})$	[82,88]
δ_{V0}	Proportion of mf per mg developing to the infective L3 stage per bite when $M_{(i)}(t) \to 0$	Dimensionless, 0·0207	[88]
c_V	Severity of constraining density-dependent larval development per dermal microfilaria	0·00878	[82]
ν_1	Per capita development rate from L1 to L2 larvae	201·6 year^{-1}	[90]
ν_2	Per capita development rate from L2 to L3 larvae	207·7 year^{-1}	[90]
μ_V	Per capita mortality rate of blackfly vectors	26 year^{-1}	[82,88]
α_V	Per capita microfilaria-induced mortality of blackfly vectors	0·39 year^{-1}	[82]
τ_V	Delay before L1 larvae can start transitioning to L2 stages	0·011 years (4 days)	[90]
Ivermectin treatment			
$\mu'_{M(i)}(\tau_{h(i)}) = (\tau_{h(i)} + u)^{-\kappa}$	Ivermectin-induced per capita rate of excess mortality of microfilariae at time $\tau_{h(i)}$ since treatment, Eq (S16)	Defined by u and κ	[89]
u	Constant to allow for very large yet finite microfilaricidal effect upon treatment with ivermectin	$9{\cdot}6\times10^{-3}$	[89]

κ	Shape parameter for excess microfilarial mortality following treatment with ivermectin	1·25	[89]
$\lambda'_{(i)}(\tau_{h(i)}) = \lambda^{max} e^{(-\varphi \tau_{h(i)})}$	Ivermectin-induced per capita rate of reversion from fertile to non-fertile adult female *O. volvulus* at time $\tau_{h(i)}$ since the last treatment, Eq (S17)	Defined by λ^{max} and φ	[89]
λ^{max}	Maximum rate of ivermectin-induced female worm sterility	32·4 year^{-1}	[89]
φ	Rate of decay of ivermectin-induced female worm sterilisation	19·6 year^{-1}	[89]
λ'_p	Proportion of adult female worms made permanently infertile at each ivermectin treatment round	0·345	[89]
**Skin microfilarial intensity (density) and prevalence**			
$M^*_{(i,k)}(t)$	The observed number of microfilariae in a single skin snip k from human host i	Model output	[81]
w	The average weight of skin for one skin snip (taken with a Holth-type corneoscleral punch [27])	2 mg	[88,91]
$\bar{M}^*_{(i)}(t)$	The mean number of microfilariae per mg skin from n skin snips of weight w in human host i	Model output	[81]
$\bar{P}^*_{(i)}(t)$	A binary variable indicating positivity for microfilariae in human host i	Model output	[81]
n	The number of skin snips taken per individual human host	2	[81]
$\bar{M}^*(t)$	The mean number of microfilariae per mg of skin per human host	Model output	[81]
$k_{M(i)} = k_{M0} + k_{M1} W_{F(i)}$	The degree of microfilarial aggregation within the skin of human host i	defined by k_{M0} and k_{M1}	[81]
k_{M0}	The degree of microfilarial aggregation in the skin as $W_{F(i)}(t) \to 0$	0·313	[81]
k_{M1}	The change in microfilarial aggregation with increasing $W_{F(i)}(t)$	0·048 per adult female worm	[81]

A6.3 Modelling approach, scenarios and mitigation strategy

A6.3.1 Pre-control mf prevalence setting

We simulated pre-control *O. volvulus* mf prevalence levels ranging from 40%-80% (i.e. from meso- to hyperendemicity) with each model. The following key transmission parameters were varied between simulations to match pre-control mf prevalence levels: 1) annual biting rate (both models), 2) variation in exposure to vector bites between individuals in the population (both models), and 3) the level of external force of infection (only ONCHOSIM). We ran the models with different parameter values and accepted combinations when the pre-control mf prevalence in the endemic equilibrium would fall into this range. Both models were run until we had 100 simulations (i.e. parameter combinations) for each 1% prevalence bin.

A6.3.2 Scenarios

Scenarios are modelled for African settings with annual MDA since 2014 (short history of control), across the whole range of pre-control mf prevalence values. The annual MDA coverage was always assumed to be 65% of the total population, with on average 5% of the population never participating in treatment (systematic non-adherence 5%). We simulated the following scenarios:

1. No interruption of annual MDA
2. 6, 12, and 18 months interruption of MDA treatment due to COVID19

Biannual treatment in the year following the 6-, 12- or 18-month interruption was simulated as a mitigation strategy. The coverage of the mitigation strategy was 65% with 5% systematic non-adherence.

All scenarios and mitigation strategies were simulated until 2030. For the interruption scenarios and mitigation strategy, we extended the simulated period to 2033, in order to assess the delays of reaching the mf prevalence level of 2030 without interruption. Delays were calculated by assessing the year when the interruption (and mitigation) scenario fell below the mf prevalence level in 2030 without interruption. The prevalence levels in the medium and high endemic setting were based on the average of all repeats (i.e. 2000 repeats) within 40-60% and 60-80% bins, respectively. Table A6.3 shows the corresponding mean delays to reach the mf prevalence in 2030 without interruption using ONCHOSIM and EPIONCHO-IBM. Figure A6.1 shows the underlying predicted mean mf prevalence dynamics during MDA.

Table A6.3. Predicted mean delays to reach the mf prevalence in 2030 without interruption.

	**Additional years required to reach the mf prevalence in 2030 without interruption**					
	**6-month interruption**		**12-month interruption**		**18-month interruption**	
**Pre-control mf prevalence (%)**	**ONCHOSIM**	**EPIONCHO-IBM**	**ONCHOSIM**	**EPIONCHO-IBM**	**ONCHOSIM**	**EPIONCHO-IBM**
40 - 60 (mesoendemic)	1	0	1	2	1	2
60 - 80 (hyperendemic)	1	1	2	3	2	3

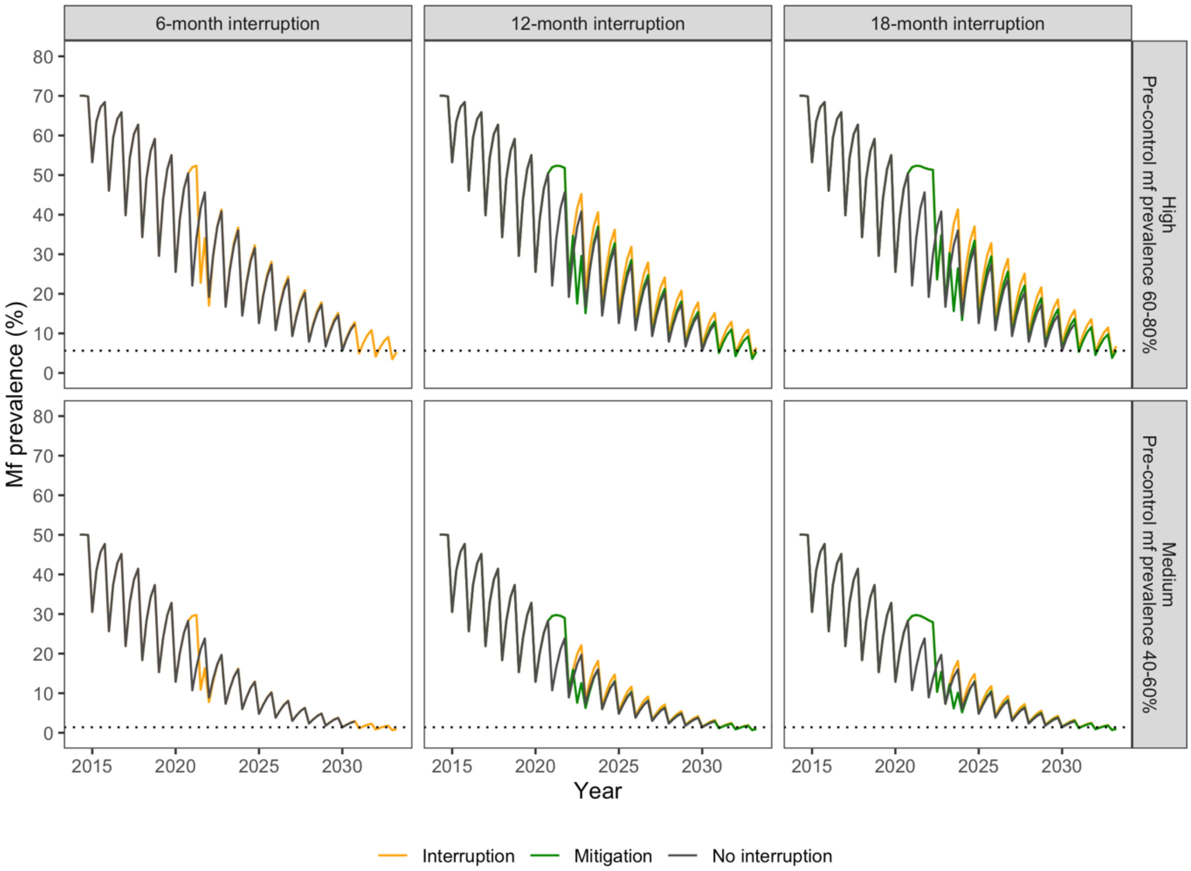

Figure A6.1. Mean microfilarial (mf) prevalence dynamics during ivermectin MDA predicted by ONCHOSIM. The colors represent the different scenarios, i.e. no interruption, interruption, and mitigation (biannual treatment). The dotted line represents the mf prevalence level in 2030 without interruption.

A7. Trachoma: Additional model details and supplementary results

A7.1 Trachoma model details and parameters

The model utilised here is an individual-based stochastic model of ocular *C. trachomatis* transmission which accounts for active trachoma (trachomatous inflammation—follicular, TF) persisting after clearance of *C. trachomatis* infection (Borlase et al., under review). This model is based on a previously described framework [93] which was validated as the most parsimonious and best fit to cross-sectional infection (PCR) and TF data in a study which compared several possible frameworks for ocular *C. trachomatis* transmission.[94]

In this framework individuals transition through four sequential states: Susceptible (S), infected but not yet diseased (I), infected and diseased (ID) and diseased but no longer infected (D), illustrated schematically in Figure A7.1. Here disease refers specifically to TF. Within this framework, people who have cleared infection but remain diseased (D) are susceptible to infection but with force of infection (λ) reduced by a factor (Γ).

Model parameters, definitions, values and sources are given in Table A7.1

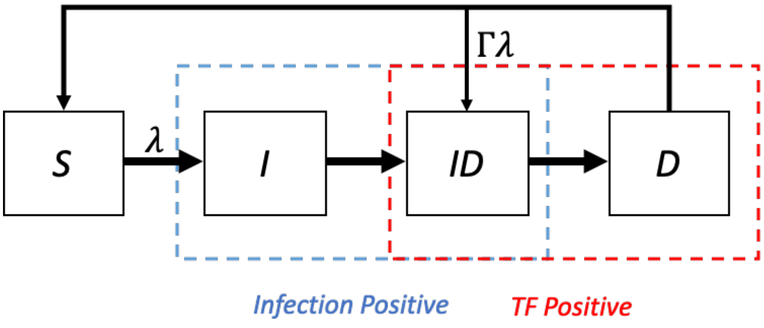

Figure A7.1 Schematic of trachoma model structure. Individuals can be susceptible to infection (S), infected but not yet diseased (I), infected and diseased (ID) or diseased but having cleared infection (D). Disease refers to trachomatous inflammation—follicular (TF). Individuals for whom infection has been cleared but disease persists (D) can be re-infected with force of infection (λ) reduced by Γ.

Table A7.1. Trachoma model variables, parameters and sources.

Notation	Description	Values /distribution	Units	Source
S_i	Susceptible individuals	-		
$I_{i,j}$	Infected and not yet diseased (individual *i* infection *j*)	14	Days	[95]
$ID_{i,1}$	Infected and diseased period (individual *i,* first infection)	$\sim Poisson(\omega_{max})$	Days	
$ID_{i,j}$	Infected and diseased period (individual *i,* infection *j*)	$(ID_{i,1} - \omega_{min})e^{\phi(j-1)} + \omega_{min}$	Days	[93–95]
$D_{i,1}$	Diseased only period (individual *i,* infection *j*)	$\sim Poisson(\tau_{max})$	Days	
$D_{i,j}$	Diseased only period (individual *i,* infection *j*)	$(D_{i,1} - \tau_{min})e^{\theta(j-1)} + \tau_{min}$	Days	[93–95]
ω	Duration of infected and diseased period	$\omega_{max} = 200, \omega_{min}$=77	Days	[93–95]
τ	Duration of diseased only period	$\tau_{max} = 300, \tau_{min}$=7	Days	[93–95]
ϕ	Decay rate, infected and diseased period	0·45	Proportion	[93–95]
θ	Decay rate, diseased only period	0·3	Proportion	[93–95]
b	Infectivity of an individual proportional to their bacterial load	0·114	Proportion	[93,94]
β	Transmission parameter	Varied to simulate a range of settings		
λ_a	Force of infection for age group *a*	Calculated	Weeks	[93,94]
Γ	Reduction in force of infection during disease only state	0·5	Proportion	[93,94,96]
c	Treatment coverage (proportion of population receiving treatment at each round of MDA)	0·8	Proportion	[97]
ϵ	Treatment efficacy (probability of infection clearance given that treatment is received)	0·85	Proportion	[98]

Following the original model and evidence from empirical studies, [93,99,100] duration of *ID* and *D* disease for each individual are assumed to decrease with each subsequent infection. For each individual *i*, the duration of first ID and D periods ($ID_{i,1}$; $D_{i,1}$) are randomly assigned from Poisson distributions, with distribution means given as the baseline (longest) duration used by Pinsent and colleagues (see Table A7.1).[93] The duration of these periods for subsequent infections are then assumed to decrease following a negative exponential to a minimum value, with decay rates and minimum durations also as given by Pinsent and colleagues.[93] Similarly, it is assumed that an individual's infectivity is proportional to their bacterial load, and that this also declines from the first infection following a negative exponential with each subsequent infection. For each individual's (*i*) infection number (*j*), the calculated durations of $ID_{i,j}$ and $D_{i,j}$ are used as fixed transition periods, in contrast to exponential transitions utilised in the previous models.

Community-wide MDA is assumed to be delivered to all ages with an 80% coverage level, in line with WHO minimum target coverage,[97] and an efficacy (the probability that an individual who receives MDA clears infection) of 85% is assumed.[98] To simulate the potentially lower efficacy of topical tetracycline eye ointment (which is routinely given to children aged less than 6 months), treatment is assumed to be 50% less effective in this age group. Treatment is assumed to be distributed randomly. Additional reductions in transmission due to other interventions (i.e. facial cleanliness and environmental improvements, which are also part of the WHO strategy for trachoma control) are not currently explicitly included in the model due to uncertainty regarding their relative impact.

A7.2. Modelling approach, scenarios and mitigation strategies.

We simulated interruption and mitigation strategies in two settings with differing baseline levels of endemicity/transmission. These were high endemicity, defined as mean baseline (before MDA) TF in children aged 1-9 years (TF_{1-9}) of 40% (range 37·5-42·5), and medium endemicity, corresponding to a mean baseline TF_{1-9} of 20% (range 17·5-22·5). We simulated the interruption to MDA to be mid-way through a planned programme, assuming a 5-year MDA programme for the high endemic setting (interruption in year 3) and a 3-year MDA programme for the medium endemic setting (interruption in year 2).

In order to ensure that the age-distribution of historical infections (and therefore infectivity, duration of infection and disease) are representative for a given level of baseline endemicity, a 40-year burn-in period was implemented for all simulations (burn-in period removed from analyses). This was followed by 16 simulated years for analysis.

To represent the different levels of baseline endemicity, we varied the transmission parameter β; this can be considered a proxy for a range of hard-to-quantify factors which facilitate transmission of ocular *C. trachomatis*, including overcrowding and lack of sanitation. We then filtered the initial sets of stochastic simulations based on the specified baseline prevalence range. To ensure simulations were representative of settings that would have been expected to reach elimination as a public health problem (EPHP) threshold of TF_{1-9} <5% threshold before 2030 with a strategy of annual district-level MDA targeting the whole community, we also filtered out simulations which did not reach TF_{1-9} <5% under the no interruption scenario.

In addition to the mitigation strategy considered in the main text (an additional round of community-wide MDA delivered 6 months after the programme restarts) we also simulated an alternative mitigation strategy in which the additional round of MDA targets only children aged 6 months to 9 years.

A7.3 Additional results

The average time to reach the EPHP threshold of TF_{1-9} <5% (calculated as the mean/median of stochastic simulations) and 95% confidence intervals (given as 95th centiles) for the range of scenarios described in the main text are given in table A7.1, in addition to the alternative mitigation strategy of an extra MDA round targeting children only.

The impact in terms of average time to reaching the EPHP threshold is very similar for both mitigation strategies. This is representative of the fact that children are effectively a core group within the model, due to the assumptions of higher bacterial loads and longer durations of infection; assumptions which reflect empirical evidence.

Table A7.2. Additional results, trachoma. Average (mean and median) years to EPHP target of TF <5% in children aged 1-9 years in high and medium endemic settings under scenarios of: No MDA interruption, MDA interruption (6 months, 12 months or 18 months) and two mitigation strategies (extra community-wide MDA, ie. All age group in the year following a 12-month interruption; extra MDA targeting only children aged 6 months to 9 years of age). 95% Confidence intervals are given as 95th centiles.

**High Endemicity (Baseline 40% TF_{1-9})**						
Scenario:	No interruption	6-month interruption; No mitigation	12-month interruption; No mitigation	18-month interruption; No mitigation	12-month interruption; Mitigation= Extra community-wide MDA round	12-month interruption; Mitigation= Extra MDA round, children aged 6 months to 9 years
Mean years to achieve EPHP (Median; 95% CI)	4·4 (4·2; 2·4-11·5)	4·65 (4·6; 2·4-11·4)	7·1 (6·3; 2·4->16[a])	6·5 (6·1; 2·4->16[a])	5·3 (5·2; 2·4->16[a])	5·4 (5·3; 2·4->16[a])
**Medium Endemicity (Baseline 20% TF_{1-9})**						
Scenario:	No interruption	6-month interruption; No mitigation	12-month interruption; No mitigation	18-month interruption; No mitigation	12-month interruption; Mitigation= Extra community-wide MDA round	12-month interruption; Mitigation= Extra MDA round, children aged 6 months to 9 years only
Mean years to achieve EPHP (Median; 95% CI	2·7 (2·6; 1·8-4·6)	3·2 (3·1; 1·8-4·3)	4·0 (3·9; 1·8-5·7)	4·4 (4·3; 1·8-5·7)	3·8 (3·8; 1·8-4·6)	3·9 (3·8; 1·8-4·9)

A8. Visceral Leishmaniasis: Additional model details and supplementary results

A8.1 Model structure

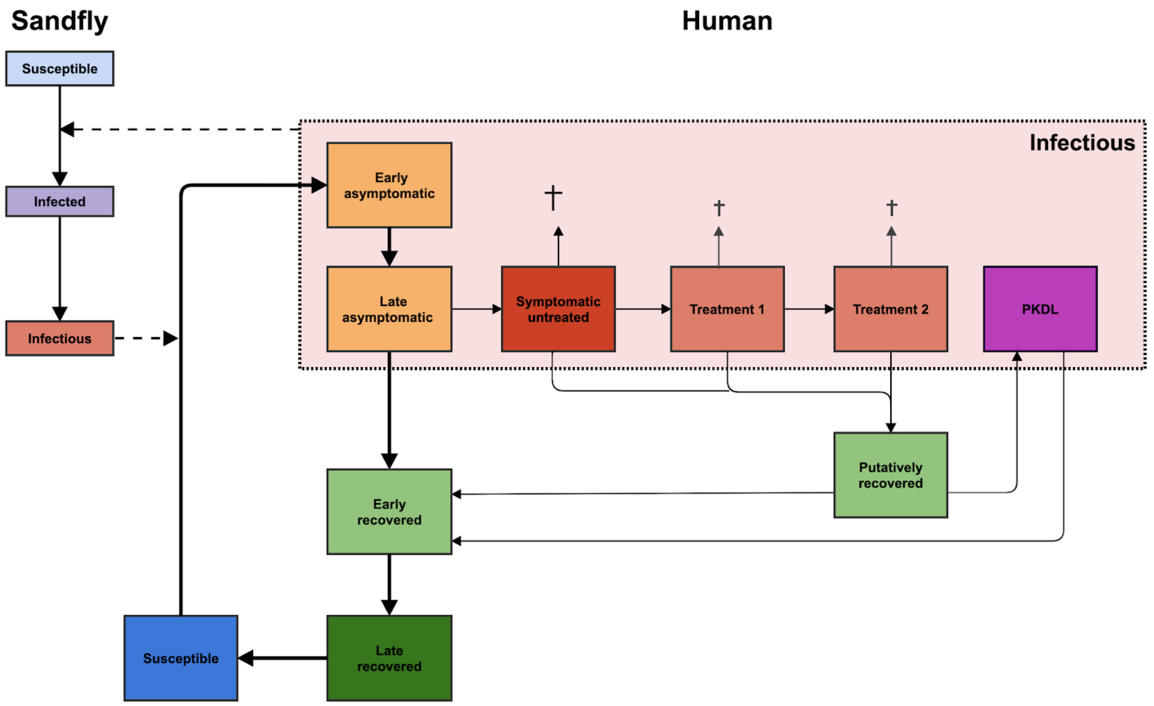

Figure A8.1. Schematic presentation of the structure of model E1 and the related model E0. For model E1, asymptomatic individuals (yellow compartments) are the main contributors to transmission. Model E0 has the same structure as model E1, but asymptomatic individuals do not contribute to transmission. Both models have different durations of infection stages from fitting to data, which are listed elsewhere.[101–104]

A8.2 Model description

The Erasmus MC models consist of a set of deterministic age-structured model variants based on different assumptions about where the main reservoir of infection lies; namely, solely in symptomatic individuals (VL and PKDL; Model E0), or mainly in asymptomatic individuals (Model E1). Other variants, with the main reservoir of infection in previously immune individuals in whom infection reactivates or PKDL cases, have also been explored.[101]

The models include population growth of both humans and sand flies (the populations are assumed to grow at the same rate in the absence of seasonality and vector control) and age-structure in human mortality and exposure to sand flies. Models E0 and E1 have a yearly seasonal pattern in sandfly density based on seasonal patterns observed in sandfly distribution studies in Bihar.[105–108] Seasonality is implemented via a stepwise function in the sand-fly birth rate, which is assumed to peak during 3 months of the year (July-September). Indoor residual spraying reduces the populations of the sandfly compartments, and active case detection leads to a shorter duration of the symptomatic untreated state (dark red) in all models. Additional details of the model, all parameter values, and calculations of equilibria of the system of ordinary differential equations along with data are provided in previous papers.[101–104]

A8.3 Model fitting

The model is calibrated based on age-structured data from approximately 21,000 individuals included in the KalaNet bednet trial in India and Nepal.[108] In the model there are compartments for early and late asymptomatic infection, and early and late recovered stage, to allow the fitting of these models to prevalence of positivity on the direct agglutination test (DAT) and/or PCR from the KalaNet study.[108]

The impact of indoor residual spraying of insecticide (IRS) was estimated using a geographical cross-validation on >5,000 VL cases from 8 endemic districts in Bihar collected by CARE India[109] for which the full model descriptions and sensitivity analyses are presented in Le Rutte, Chapman *et al*., 2017.[102]

A8.4 Bounce-back

We simulated a highly endemic setting with a pre-control equilibrium of 10 VL cases per 10,000 population per year.

During year 0 we implemented 1 year of attack phase interventions:

- vector control, IRS effect = 0·67
- active case detection (ACD), onset of symptoms to diagnosis = 45 days

From the start of year 1 onwards we simulated a situation comparable to the pre-control situation

- IRS effect = 0
- onset of symptoms to diagnosis = 60 days

The outcomes of both Model E0 and model E1 are presented in Figure A8.2, whereas Figure A2.1 includes the predictions from Model E1 only.

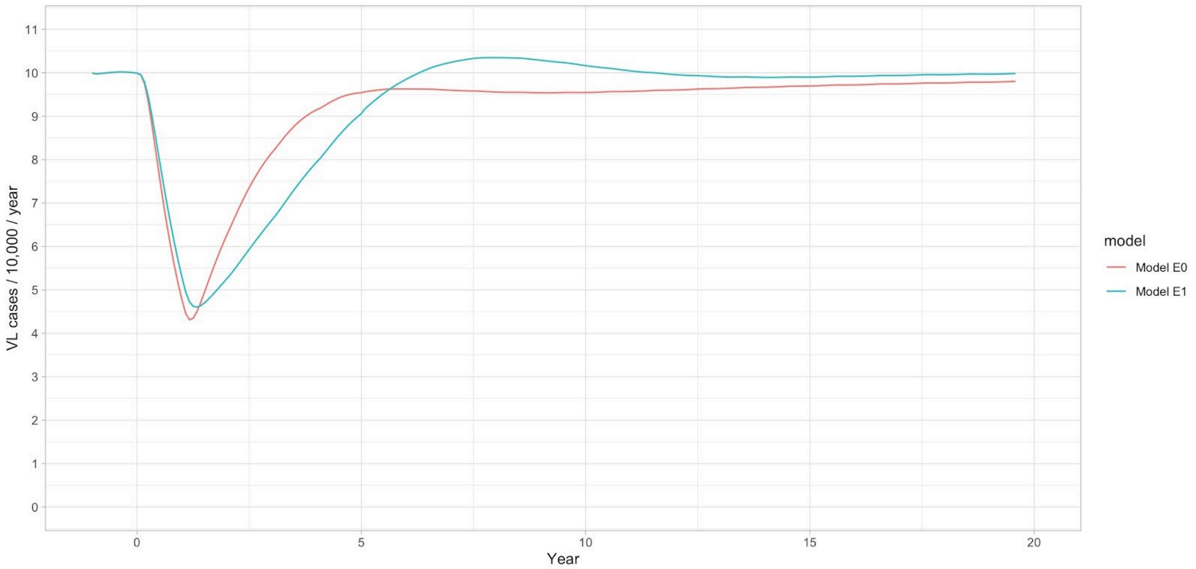

Figure A8.2. Bounce-back trajectories of VL incidence following one year of intervention at baseline for models E0 and E1.

A8.5 Delays to the target

We also simulated VL programme interruptions of 6, 12, and 18-months (besides the 12 months that are presented in the main text) for both a highly- and moderately-endemic setting using transmission Models E0 and E1. The number of years to get to the target incidence of <1/10,000/year are presented in Table A8.1, as well as the delay in years when compared to the scenario without an interruption due to Covid-19. For these scenarios we also simulated the potential impact on reducing the number of years to reach the target after implementing mitigation strategies (Table A8.2). We simulated a duration of the mitigation strategy (extended attack phase) to be equal to the duration of the interruption (6, 12 or 18 months).

Table A8.1. Years to target (delays in years) without mitigation strategy.

**VL**	**Years to target (delays in years)**			
	**Highly endemic setting**		**Moderately endemic setting**	
**Interruptions:**	**Model E0**	**Model E1**	**Model E0**	**Model E1**
**None**	14·8 (0)	9·5 (0)	2·5 (0)	2·3 (0)
**6 months***	15·4 (0·6)	10·2 (0·7)	4·1 (1·5)	3·3 (1·0)
**12 months**$**	16·2 (1·4)	10·9 (1·4)	4·8# (2·3)	4·1 (1·8)
**18 months*****	17·0 (2·2)	11·8 (2·3)	7·0 (4·5)	4·8 (2·5)

* 6 months = no IRS and ACD between 1 April 2020 and 30 September 2020
** 12 months = no IRS and ACD between 1 April 2020 and 31 March 2021
*** 18 months = no IRS and ACD between 1 April 2020 and 30 September 2021
$ The 12 months interruption scenario is presented in Table 3 of the main text.

Table A8.2. Years to target (delays in years) with mitigation strategy. The duration of the mitigation strategy is equally long as the duration of the interruption.

**VL**	**Years to target (delays in years)**			
	**High prevalence**		**Medium prevalence**	
**Interruptions:**	**Model E0**	**Model E1**	**Model E0**	**Model E1**
**None**	14·8 (0)	9·5# (0)	2·5 (0)	2·3 (0)
**6 months***	15·3 (0·1)	9·7 (0·2)	4·1(1·6)	3·3 (1·0)
**12 months**$**	15·8 (1·0)	10·0 (0·5)	4·8 (2·3)	4·1 (1·8)
**18 months*****	16·3 (1·5)	10·4 (6·1)	5·5 (3·0)	4·8 (2·5)

* 6 months = no IRS and ACD between 1 April 2020 and 30 September 2020
** 12 months = no IRS and ACD between 1 April 2020 and 31 March 2021
*** 18 months = no IRS and ACD between 1 April 2020 and 30 September 2021
$The 12 months interruption scenario is presented in Table 3 of the main text.

A8.6 Timelines of delay to the elimination target

Table A8.3. Overview of simulated scenarios. All interruption scenarios include simulations both with and without mitigation strategy, where the mitigation strategy has the same duration as the interruption.

	**Model E0**	**Model E1**
**Highly endemic setting (10/10,000/year)**	No interruption	No interruption
	6-month interruption	6-month interruption
	12-month interruption	12-month interruption*
	18-month interruption	18-month interruption
**Moderately endemic setting (5/10,000/year)**	No interruption	No interruption
	6-month interruption	6-month interruption
	12-month interruption	12-month interruption
	18-month interruption	18-month interruption

*The 12-month interruption of Model E1 for the highly endemic setting is the scenario that is presented in Figure 2 of the main text.

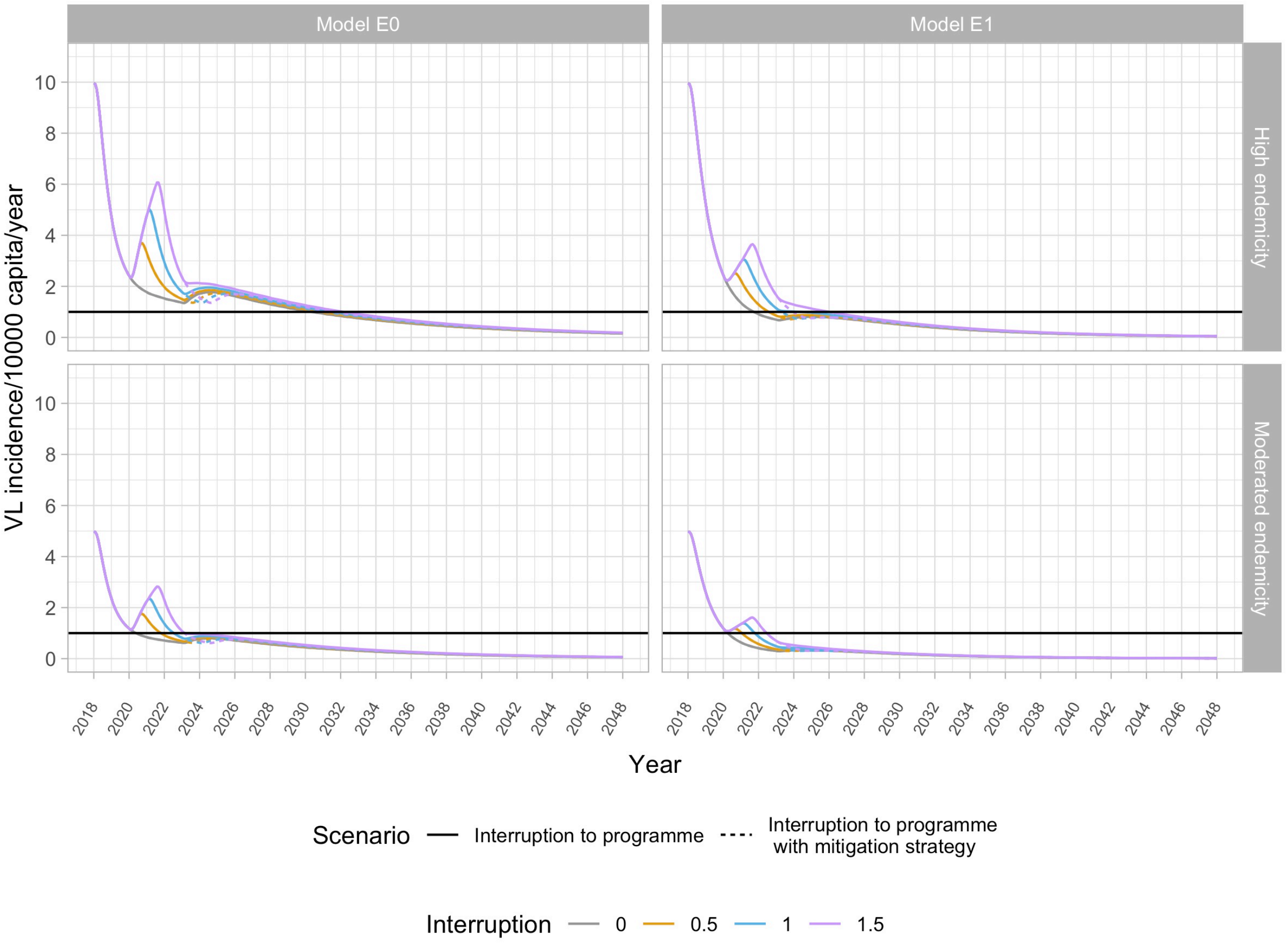

Figure A8.3. Predicted visceral leishmaniasis incidence over time. The solid lines present the impact of the interruption (in years) of VL control measures due to Covid-19 during the attack phase. The dotted lines present the predicted incidence when implementing a mitigation strategy. The grey lines represent the default scenario without an interruption of the programme. Interruptions to the programme are simulated to last from 1 April 2020 to 31 March 2021.

For interruptions at different stages of the control programme (both during the attack as well as the consolidation phase) we would like to refer to the following paper titled "**The simulated impact of COVID-19 related programme interruptions on visceral leishmaniasis in India**", by Epke Le Rutte, Luc Coffeng, Johanna Muñoz and Sake de Vlas (*soon to be linked to MedRXiv/journal*). In this paper we also present the impact on cumulative VL incidence that is caused by the interruption of the programmes besides the delay to the target.

A9. gHAT: Additional model details and supplementary results

A9.1 Modelling approach, settings and interruption scenarios simulated

Main strategies against gHAT in the DRC consist of case detection via active screening (AS) and passive surveillance (PS), with vector control (VC) implemented in the last years in a reduced number of settings. In the present study, we focused on three potential interruption scenarios of gHAT activities in DRC settings due to COVID-19. We chose regions where VC has not yet been implemented and explored the impact of altered control interventions for 6, 12, and 18 months starting on 1st April 2020. We focus here on "medium-risk" and "high-risk" settings with average levels of AS before and after the interruption. In all cases, interruption periods were simulated considering full interruption of screening activities in addition to a reduced passive surveillance (consisting of passive detection set back to pre-2000 level). Mitigation scenarios were simulated such that after interruption, active screening was resumed at maximum historical level, and passive detection set back to values before interruption.

Two previously published deterministic models (Model S and Model W) were used to perform this analysis. Both were originally calibrated to different human case data in the Democratic Republic of Congo (DRC) [110,111], which has around 70% of the case burden in 2019.[112] Both models explicitly include tsetse and have a high/low-risk structure for human

exposure to tsetse and participation in AS. Neither model includes animal reservoirs or importation. Model S simulated 10% annual AS, and Model W simulated 17% AS. PS rates for non-interruption years were inferred through the fitting. AS was assumed to take place at the beginning of each non-interruption year, whereas PS occurs throughout the year. The different level of reported cases from both data sets used by each model, and inferred transmission levels, were used to define medium-risk (Model W) and high-risk (Model S) settings. Additional information is found in model description sections 3 and 4.

A9.2 Additional results

This section includes complementary results to those presented in the main text.

A9.2.1. Years to elimination of transmission

Table A9.1 presents expected timeline (years) to elimination of transmission for medium and high-risk settings under different interruption, and interruption plus mitigation scenarios. Years are counted since 2018. Median and 95% CI are indicated.

Table A9.1. Years to elimination of transmission of gHAT.

Scenario	Medium risk setting	High-risk setting
No interruption	13 (5-27)	32 (26-43)
6-month interruption	13 (6-28)	32 (26-43)
6-month interruption plus mitigation	11 (5-21)	31 (24-41)
12-month interruption	14 (6-28)	33 (27-43)
12-month interruption plus mitigation	12 (6-22)	31 (25-41)
18-month interruption	14 (6-29)	33 (27-43)
18-month interruption plus mitigation	12 (6-22)	25-42)

A9.2.2. New infections under different scenarios

Timelines comparing the impact on new infections of different scenarios (interruption and interruption plus mitigation strategy) analysed to a scenario with no interruption.

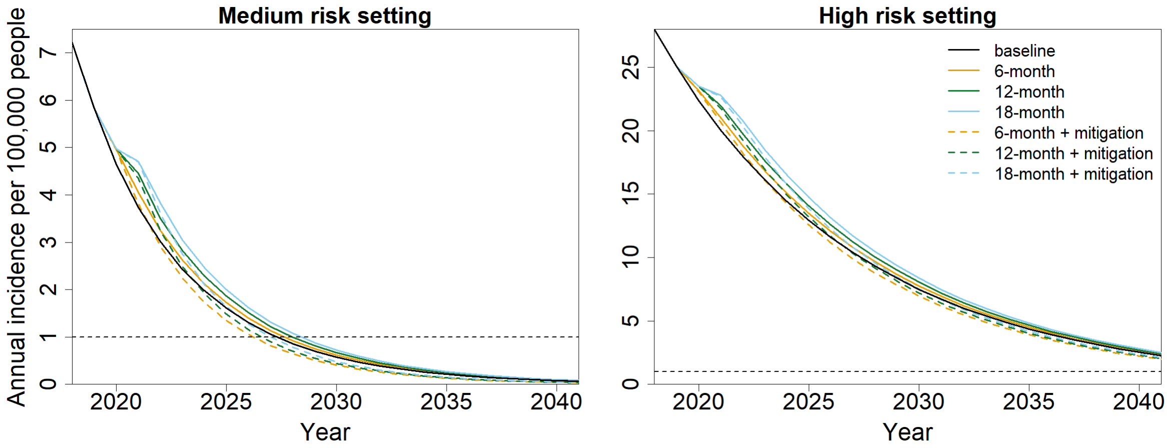

Figure A9.1. Median annual incidence. Comparing interruption and mitigation strategies to a scenario with no interruption, for medium and high-risk settings. Dashed line indicates threshold for elimination of transmission.

A9.3 Model S

A9.3.1 Description of transmission model and control interventions

The deterministic Model S used here was presented and described in (1) and is a variant of the gHAT transmission model originally published in.[113] The model consists of a system of coupled ordinary differential equations (ODEs), with compartments for tsetse, animal and human populations. These three different host types are modelled for two different settings corresponding to a low transmission area (e.g. the village, L) and a high transmission area (such as river banks or plantations, H) that enable accounting for heterogeneity in exposure to tsetse bites. The population size for tsetse, animal or humans in each setting i (i = {L, H}) is assumed to be stable by allowing the associated birth terms to compensate deaths in all the compartments. Tsetse and animal populations always stay within their setting (for example, tsetse in low transmission settings always remain in the low transmission setting and animals in high transmission settings always remain in the high transmission setting). Similarly, humans in low transmission settings always remain in low transmission setting. However, humans in the high transmission setting move back and forth between the high and low transmission settings spending a fixed amount of time in each one (to model, for example, the movement of high-risk individuals between villages and plantations) — as shown in Figure A9.2.

Five compartments describe humans in any of the two settings: susceptible (S_{hi}); exposed or incu- bating (E_{hi}); infected with the first stage of the disease (I_{h1i}); infected with the second stage of the disease, where trypanosomes have reached the cerebro-spinal fluid (I_{h2i}); and treated (T_{hi}). The total human population in setting *i* is $N_{hi} = S_{hi} + E_{hi} + I_{h1i} + I_{h2i} + T_{hi}$. Tsetse populations are divided into susceptible (S_{vi}); teneral (U_{vi}); exposed (E_{vi}); and infected (I_{vi}), so that the vector population is $N_{vi} = S_{vi} + U_{vi} + E_{vi} + I_{vi}$.

In this model implementation: *i)* animals do not contribute to transmission, thus animal populations are modelled as constant parameters, N_{ai}, and only form a sink for tsetse bite; *ii)* both stages (rather than only stage 1) of the disease are exposed to tsetse fly bites; *iii)* an additional compartment in the vector dynamics, U_i, accounts for the teneral effect — a reduction of infectivity with time — such that on average tsetse are only infectious for the first five days after emergence. A schematic of the model is shown in Figure A9.2.

Test and treat interventions encompass both active screening and passive surveillance. Passive detection is represented by a continuous stage-specific detection rate and removes infected people from both low- and high-risk settings whilst active screening only recruits people in the low risk setting. With the available staged data suggesting an enhanced passive surveillance system, an improvement with time was included in the detection rate of stage 2, r2 by multiplying the fitted constant of proportionality, c2, by the proportion of people screened through passive surveillance as informed by data which showed an increasing trend.

We followed [114] to relate a proportion, d, of humans effectively screened in a given a year and the daily removal rate $r_{as}^{continuous}$ as $d = 1 - \exp\left(-365 r^{continous}\right)$.

Thus, for the pulsed active screening, we get: $r_{as}^{pulsed} = r_{as}^{continuous} = -\left(\frac{12}{365}\right)\ln\left(1 - d\right)$.

Screening levels were informed from data; estimates for the population of Bandundu were taken from [115] for the period corresponding to calibration, and a 3% annual growth was assumed for projections.

The unknown proportion of the population at risk of infection in Bandundu province is included via ϵ, such that

$d(t0 = \frac{X_S(t)}{\epsilon N_B(t)}$, where $X_s(t)$ indicates number of people screened in year t, and $N_B(t)$ indicates Bandundu province population in year t. With no additional data enabling estimating ϵ, this parameter was set as a constant value.

A9.3.2 Parameter values

Except ϵ and α which are parameters new to the model and that were assumed fixed since their incorporation in (1), model parameters assigned fixed were taken from Model S posteriors (median) in [116], and are described in Table A9.2.

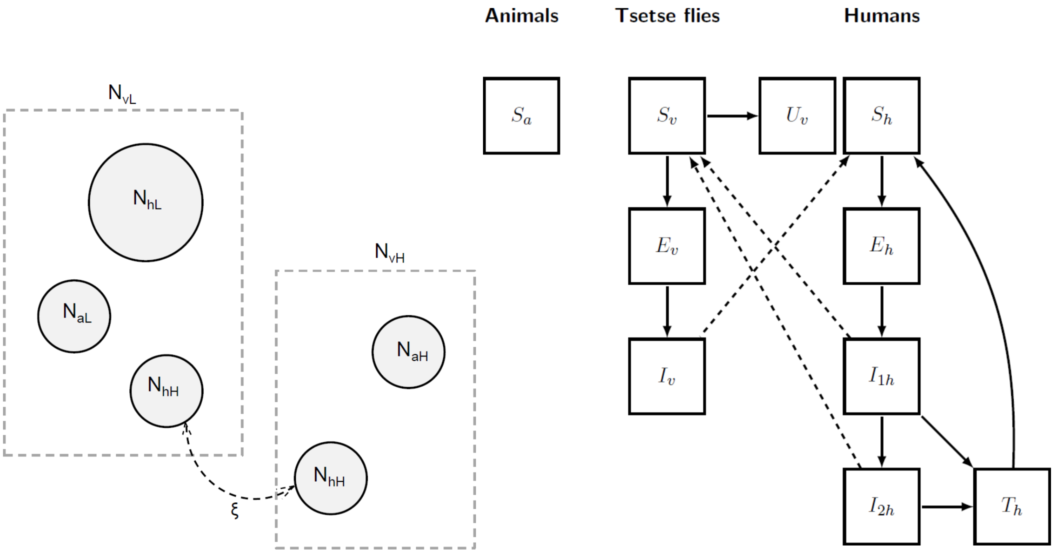

113.

Table A9.2. Model parameterisation (fixed parameters). Notation, a brief description, and the used values of fixed parameters in Model S.

Notation	Description	Value
α	Rate at which tsetse become non-teneral (i.e. cannot get infectious)	73 $year^{-1}$
A/H_1	Density of animals relative to humans in area *L*	0·7
A/H_2	Density of animals relative to humans in area *H*	0·9
b	Proportion of infective bites leading to infection in humans and animals	0·8
c_{ai}	Proportion of bites on an infective animal of type *i* that lead to a mature infection in flies	0
δ	Rate at which treated humans return to the susceptible class	2·19 $year^{-1}$
δ_a	Rate of loss of immunity in animal hosts	1·095 $year^{-1}$
ϵ	Proportion of population at risk	0·7
η	Rate at which hosts move from the incubating stage	31·025 $year^{-1}$
f	Inverse of duration of feeding cycle; or biting rate	121·545 $year^{-1}$
γ	Rate of progression to stage 2 in humans	0·365 $year^{-1}$
γ_{aL}	Rate of progression to the immune class in animal hosts of type *L*	0·73 $year^{-1}$
γ_{aH}	Rate of progression to the immune class in animal hosts of type *H*	0·6935 $year^{-1}$
μ	Death rate of humans due to natural causes	0·02 $year^{-1}$
μ_{ai}	Death rate of animal host of type *i*	0·511 $year^{-1}$
μ_t	Death rate of humans due to treatment	0 $year^{-1}$
μ_v	Death rate of tsetse	10·95 $year^{-1}$
ν	Inverse of the extrinsic incubation period	13·505 $year^{-1}$
r_1	Removal rate of infected humans in stage 1 due to treatment (passive detection)	4·6144 $year^{-1}$
σ	Biting preference for humans	0·4
σ_{ai}	Biting preference for animal in the setting i	0·3
ξ	Proportion of time spent i the high-risk region by commuters	0·62

A9.3.2 Summary of previous fitting

The deterministic ODE version of Model S was calibrated to province level data for Bandundu (Democratic Republic of Congo) using an Aproximate Bayesian Computation (ABC) algorithm in a previous work 110 fitting six parameters. The data consisted of annual, staged reported cases for 2000-2012 from active screening and passive detection (indicated as fit to ”staged data' in [110]). A summary of the fitted parameter posteriors is given in Table A9.3.

Table A9.3. Model parameterisation (posterior parameters). Notation, a brief description, the median values, and the 95% certainty intervals of fitting parameters in Model S.

Notation	Description	Median	Value 95% CI	Unit
c_h	Proportion of bites on an infective human that lead to a mature	3·2134 $\times 10^{-3}$	[2·554, 3·9612] ($\times 10^{-3}$)	-
	**Infection in flies**			
c_2	Constant of proportionality relating proportion of population screened to detection rate	22·3726	[15·2063, 33·0127]	[$year^{-1}$]
μ_γ	Disease-induced death rate or rate of leaving the recovered state for humans	0·6189	[0·4852, 0·7261]	[$year^{-1}$]
m_{HL}	Ratio of humans in the high-exposure to low-exposure environment	0·2468	[0·1508, 0·2951]	-
vh_L	Number of vectors per human in area *L*	3·1426	[2·4479, 3·9298]	-
vh_H	Number of vectors per human in area *H*	3·6195	[2·8221, 4·4467]	-

A9.4 Model W

A9.4.1 Description

The original model [111] describes dynamics of gHAT transmission explicitly considering compartments of humans and tsetse. Figure A9.3 shows a schematic description of gHAT dynamics in this model. Humans can be exposed and subsequently infectious by a bite of an infectious tsetse. They progress through different stages of the infection (stage 1 and stage 2) with different rates (σ_H and ϕ_H respectively). On the other side, tsetse vectors can become exposed and subsequently infectious if they bite an infectious human. Infected people may be detected by passive and active screening, followed by hospitalisation and recovery. Here, we consider a version of the model where humans are partitioned into two compartments of (i) low-risk and participating in the active screening, and (ii) high-risk and non-participating in active screening. We assume there are no animal reservoirs although animals receive some proportion of tsetse bites. For simplicity, we assume the total population of humans to be constant, however, we take into account growth of population (3%) for comparison to the observed data. This model accounts for the possibility of detecting of infected humans through passive and active screening. Passive screening describes potential visits of people to fixed medical centers for testing. Before 1998 (pre-active screening) it was assumed that passive detection was less effective than after activities began, and only so identified stage 2 individuals at a rate γ_H^{pre} which is smaller than the stage 2 passive detection rate from 1998 onwards, γ_H^{post}.

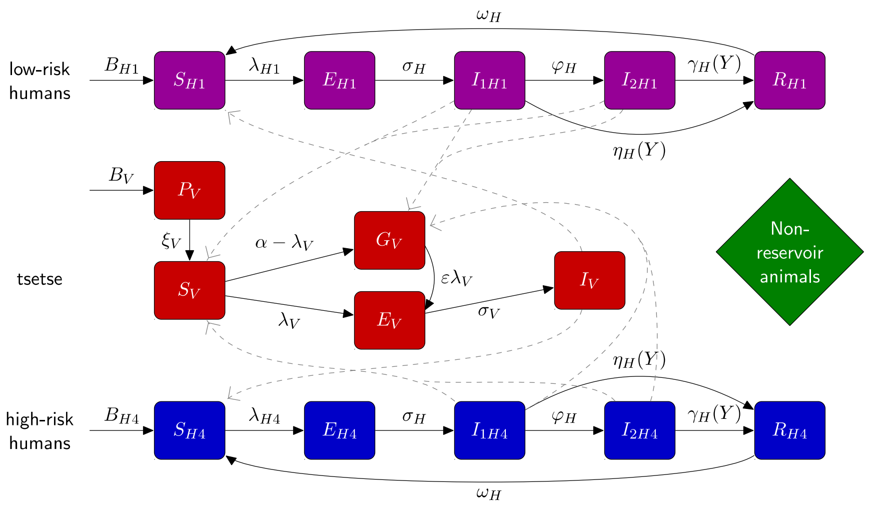

Figure A9.3. Schematic of Model W to describe gHAT infection dynamics. This multi-host model of gHAT takes into account high- and low-risk groups of humans and their interactions with tsetse vectors. Each group consists of different compartments: Susceptible humans S_{Hi} can become exposed on a bite of an infectious tsetse. Exposed people E_{Hi} progress to become the stage 1 infected people and eventually stage 2 (if not detected in active screening), and once treated they recover by hospitalization R_{Hi}. Active screening can accelerate treatment rate of infected people. Here we assume high-risk group does not participate in active screening. By biting an infectious person, tsetse can become exposed and subsequently infectious, E_V and I_V. G_V represents the tsetse population not exposed to *Trypanosoma brucei gambiense* in the first blood-meal and are therefore less susceptible in the following meals. Rates are shown by Greek letters associated with arrows. Animal reservoir is not considered. This figure is taken from [111] and adapted from the original model schematic [117].

Following previous modelling work using gHAT data from former Bandundu province[110], there is a strong signal from epidemiological staging data that passive screening has improved during the time period from 2000–2016. To capture the steadily increasing trend in the proportion of stage 1 to stage 2 passive detections, the model utilises the following formulae:

$$\eta_H(Y) = \eta_H^{post}\left[1 + \frac{\eta_{Hamp}}{1 + \exp\left(-d_{steep}(Y - d_{change})\right)}\right]$$

$$\gamma_H(Y) = \gamma_H^{post}\left[1 + \frac{\gamma_{Hamp}}{1 + \exp\left(-d_{steep}(Y - d_{change})\right)}\right]$$

where Y is the year and η_H is the stage 1 passive detection rate and γ_H is the stage 2 passive detection rate. Parameters dictating the amplitude, steepness and switching year can be found in Tables A9.4 and A9.5. Four parameters, d_{change}, η_{Hamp},

γ_{Hamp}, and d_{steep}, describing the change of passive detection over time have been estimated through fitting to the health-zone-level data for Mosango.

Similar to the previous models, we allow for the imperfect nature of the tests by considering sensitivity of tests to detect true cases and specificity to observe false positive cases. Specificity is set to one after 2015 due to improvement in confirmatory quality control.[111]

Using a similar approach to the previous ODE models, we consider the same level of screening as reported between 2000–2016. It is assumed, as in much of the previous published studies using this model, that active screening began in 1998 and achieved the same number of people screened as in 2000 (the first year of data). After 2016 and before the COVID interruption, we use the average number of screened people between 2012–2016 in all scenarios.

A9.4.2 Parameter values

As in previous versions of Model W [110,116–119], some parameters with estimates available in the literature were assigned fixed values. Fixed values are given in Table A9.4. The other parameter values were taken from posterior distributions by fitting the model to data (see 4.3 for an outline of methods and summary of statistics of parameters).

Table A9.4. Model parameterisation (fixed parameters). Notation, a brief description, and the used values of fixed parameters in Model W.

Notation	Description	Value	
N_H	Total human population size (in 2015)	121,433	(11) [120]
B_H	Total human birth rate	$= \mu_H N_H$	
μ_H	Natural human mortality rate	$5{\cdot}4795\times10^{-5}$ days^{-1}	[121]
σ_H	Human incubation rate	$0{\cdot}0833$ days^{-1}	[122]
ϕ_H	Stage 1 to 2 progression rate	$0{\cdot}0019$ days^{-1}	[123,124]
ω_H	Recovery rate/waning-immunity rate	$0{\cdot}006$ days^{-1}	[125]
Sens	Active screening diagnostic sensitivity	0·91	[126]
B_V	Tsetse birth rate	$0{\cdot}0505$ days^{-1}	[118]
ξ_V	Pupal death rate	$0{\cdot}037$ days $^{-1}$	
K	Pupal carrying capacity	$= 111{\cdot}09 N_H$	[118]
P(pupating)	Probability of pupating	0·75	
μ_V	Tsetse mortality rate	$0{\cdot}03$ days^{-1}	[122]
σ_V	Tsetse incubation rate	$0{\cdot}034$ days^{-1}	[127,128]
α	Tsetse bite rate	$0{\cdot}333$ days^{-1}	[129]
p_V	Probability of tsetse infection per single infective bite	0·065	[122]
ε	Reduced non-teneral susceptibility factor	0·05	[117]
f_H	Proportion of blood-meals on humans	0·09	[130]
$disp_{act}$	Overdispersion parameter for active detection	4×10^{-4}	[111]
$disp_{pass}$	Overdispersion parameter for passive detection	$2{\cdot}8 \times 10^{-5}$	[111]

A9.4.3 Summary of previous fitting

Model W was fitted to health-zone-level data for Mosango using an adaptive Metropolis-Hastings MCMC algorithm [111]. A summary of the fitted parameter posteriors is given below.

Table A9.5. Model parameterisation (posterior parameters). Notation, a brief description, the median values, and the 95% credible intervals of fitted parameters in Model W.

Notation	Description	Value Median	95% CI	Unit
R_0	Basic reproduction number (NGM approach)	1·012	[1·007,1·026]	-
r	Relative bites taken on high-risk humans	3·241	[1·683,6·530]	-
k_1	Proportion of low-risk people	0·9267	[0·7985,0·9787]	-
k_4	Proportion of high-risk people	$k_4 = 1 - k_1$		-
η_H^{post}	Treatment rate from stage 1, 1998 onwards	$1{\cdot}064\times10^{-4}$	$[0{\cdot}361,2{\cdot}517]\times10^{-4}$	days^{-1}
γ_H^{post}	Treatment rate from stage 2 (1998 onwards)	$2{\cdot}542\times10^{-3}$	$[1{\cdot}152,6{\cdot}173]\times10^{-3}$	days^{-1}
$b_{\gamma_H^{pre}}$	Relative treatment rate from stage 2 factor, pre-1998	0·7908	[0·6487,0·9819]	-
γ_H^{pre}	Treatment rate from stage 2, pre-1998	$\gamma_H^{pre} = b_{\gamma_H^{pre}}\ \gamma_H^{post}$		days^{-1}
Spec	Active screening diagnostic specificity	0·9992	[0·9987,0·9997]	-
u	Proportion of passive cases reported	0·3289	[0·2376,0·4328]	-
d_{change}	Midpoint year for passive improvement	2004·9	[2002·7,2010·0]	Year
$\eta_{H\text{amp}}$	Relative improvement in passive stage 1 detection rate	1·035	[0·179,3·579]	-
$\gamma_{H\text{amp}}$	Relative improvement in passive stage 2 detection rate	0·3250	[0·0370,0·9194]	-
d_{steep}	Speed of improvement in passive detection rate	1·037	[0·737,1·387]	years^{-1}

A10 References

1 Anderson RM, May RM. Population dynamics of human helminth infections: control by chemotherapy. *Nature* 1982; **297**: 557–63.

2 Anderson RM, May RM. Helminth Infections of Humans: Mathematical Models, Population Dynamics, and Control. 1985: 1–101.

3 Coffeng LE, Bakker R, Montresor A, de Vlas SJ. Feasibility of controlling hookworm infection through preventive chemotherapy: a simulation study using the individual-based WORMSIM modelling framework. *Parasit Vectors* 2015; **8**: 541.

4 Truscott JE, Turner HC, Farrell SH, Anderson RM. Soil-Transmitted Helminths. 2016: 133–98.

5 Farrell SH, Coffeng LE, Truscott JE, *et al.* Investigating the Effectiveness of Current and Modified World Health Organization Guidelines for the Control of Soil-Transmitted Helminth Infections. *Clin Infect Dis* 2018; **66**: S253–9.

6 Jambulingam P, Subramanian S, de Vlas SJ, Vinubala C, Stolk WA. Mathematical modelling of lymphatic filariasis elimination programmes in India: required duration of mass drug administration and post-treatment level of infection indicators. *Parasit Vectors* 2016; **9**: 501.

7 Bradley M, Chandiwana SK, Bundy DAP, Medley GF. The epidemiology and population biology of Necator americanus infection in a rural community in Zimbabwe. *Trans R Soc Trop Med Hyg* 1992; **86**: 73–6.

8 Elkins DB, Haswell-Elkins M, Anderson RM. The epidemiology and control of intestinal helminths in the Pulicat Lake region of Southern India. I. Study design and pre- and post-treatment observations on Ascaris lumbricoides infection. *Trans R Soc Trop Med Hyg* 1986; **80**: 774–92.

9 Bundy DAP, Cooper ES, Thompson DE, Anderson RM, Didier JM. Age-related prevalence and intensity of Trichuris trichiura infection in a St. Lucian community. *Trans R Soc Trop Med Hyg* 1987; **81**: 85–94.

10 Truscott JE, Ower AK, Werkman M, *et al.* Heterogeneity in transmission parameters of hookworm infection within the baseline data from the TUMIKIA study in Kenya. *Parasit Vectors* 2019; **12**: 442.

11 Means AR, Ajjampur SSR, Bailey R, *et al.* Evaluating the sustainability, scalability, and replicability of an STH transmission interruption intervention: The DeWorm3 implementation science protocol. *PLoS Negl Trop Dis* 2018; **12**: e0005988.

12 Bethony J, Brooker S, Albonico M, *et al.* Soil-transmitted helminth infections: ascariasis, trichuriasis, and hookworm. *Lancet* 2006; **367**: 1521–32.

13 Anderson R, Truscott J, Hollingsworth TD. The coverage and frequency of mass drug administration required to eliminate persistent transmission of soil-transmitted helminths. *Philos Trans R Soc B Biol Sci* 2014; **369**: 20130435.

14 Truscott JE, Hollingsworth T, Brooker SJ, Anderson RM. Can chemotherapy alone eliminate the transmission of soil transmitted helminths? *Parasit Vectors* 2014; **7**: 266.

15 Croll NA, Anderson RM, Gyorkos TW, Ghadirian E. The population biology and control of Ascaris lumbricoides in a rural community in Iran. *Trans R Soc Trop Med Hyg* 1982; **76**: 187–97.

16 Hotez PJ, Brooker S, Bethony JM, Bottazzi ME, Loukas A, Xiao S. Hookworm Infection. *N Engl J Med* 2004; **351**: 799–807.

17 Brooker S, Bethony J, Hotez PJ. Human Hookworm Infection in the 21st Century. 2004: 197–288.

18 Coffeng LE, Truscott JE, Farrell SH, *et al.* Comparison and validation of two mathematical models for the impact of mass drug administration on Ascaris lumbricoides and hookworm infection. *Epidemics* 2017; **18**: 38–47.

19 Turner HC, Truscott JE, Bettis AA, Hollingsworth TD, Brooker SJ, Anderson RM. Analysis of the population-level impact of co-administering ivermectin with albendazole or mebendazole for the control and elimination of Trichuris trichiura. *Parasite Epidemiol Control* 2016; **1**: 177–87.

20 Anderson RM, Schad GA. Hookworm burdens and faecal egg counts: an analysis of the biological basis of variation. *Trans R Soc Trop Med Hyg* 1985; **79**: 812–25.

21 Augustine DL. Investigations on the control of hookworm disease. XVI. Length of life of hookworm larvae from the stools of different individuals. *Am J Epidemiol* 1923; **3**: 127–36.

22 Levecke B, Montresor A, Albonico M, *et al.* Assessment of Anthelmintic Efficacy of Mebendazole in School Children in Six Countries Where Soil-Transmitted Helminths Are Endemic. *PLoS Negl Trop Dis* 2014; **8**: e3204.
23 Pullan RL, Kabatereine NB, Quinnell RJ, Brooker S. Spatial and Genetic Epidemiology of Hookworm in a Rural Community in Uganda. *PLoS Negl Trop Dis* 2010; **4**: e713.
24 Easton A V., Oliveira RG, Walker M, *et al.* Sources of variability in the measurement of Ascaris lumbricoides infection intensity by Kato-Katz and qPCR. *Parasit Vectors* 2017; **10**: 256.
25 World Health Organization. Helminth control in school-age children: A guide for managers of control programmes. 2011.
26 Anderson RM, Turner HC, Farrell SH, Truscott JE. Studies of the Transmission Dynamics, Mathematical Model Development and the Control of Schistosome Parasites by Mass Drug Administration in Human Communities. 2016: 199–246.
27 Toor J, Turner HC, Truscott JE, *et al.* The design of schistosomiasis monitoring and evaluation programmes: The importance of collecting adult data to inform treatment strategies for Schistosoma mansoni. *PLoS Negl Trop Dis* 2018; **12**: e0006717.
28 Graham M, Ayabina D, Lucas TCD, Collyer BS, Medley GF, Hollingsworth TD TJ. SCHISTOX: An individual based model for the epidemiology and control of schistosomiasis. .
29 Truscott JE, Gurarie D, Alsallaq R, *et al.* A comparison of two mathematical models of the impact of mass drug administration on the transmission and control of schistosomiasis. *Epidemics* 2017; **18**: 29–37.
30 Toor J, Rollinson D, Turner HC, *et al.* Achieving Elimination as a Public Health Problem for Schistosoma mansoni and S. haematobium: When Is Community-Wide Treatment Required? *J Infect Dis* 2020; **221**: S525–30.
31 De Vlas SJ, Gryseels B, Van Oortmarssen GJ, Polderman AM, Habbema JDF. A model for variations in single and repeated egg counts in schistosoma mansoni infections. *Parasitology* 1992; **104**: 451–60.
32 De Vlas SJ, Nagelkerke NJD, Habbema JDF, van Oortmarssen GJ. Statistical models for estimating prevalence and incidence of parasitic diseases. *Stat Methods Med Res* 1993; **2**: 3–21.
33 Chan MS, Guyatt HL, Bundy DAP, Booth M, Fulford AJC, Medley GF. The development of an age structured model for schistosomiasis transmission dynamics and control and its validation for Schistosoma mansoni. *Epidemiol Infect* 1995; **115**: 325–44.
34 Turner HC, Truscott JE, Bettis AA, *et al.* Evaluating the variation in the projected benefit of community-wide mass treatment for schistosomiasis: Implications for future economic evaluations. *Parasit Vectors* 2017; **10**: 213.
35 Fulford AJC, Butterworth AE, Ouma JH, Sturrock RF. A statistical approach to schistosome population dynamics and estimation of the life-span of Schistosoma mansoni in man. *Parasitology* 1995; **110**: 307–16.
36 Zwang J, Olliaro PL. Clinical Efficacy and Tolerability of Praziquantel for Intestinal and Urinary Schistosomiasis—A Meta-analysis of Comparative and Non-comparative Clinical Trials. *PLoS Negl Trop Dis* 2014; **8**: e3286.
37 World Health Organization Expert Committee. Prevention and control of schistosomiasis and soil-transmitted helminthiasis. World Health Organization Technical Report Series. 2002.
38 Prada JM, Davis EL, Touloupou P, *et al.* Elimination or resurgence: modelling lymphatic filariasis after reaching the 1% microfilaremia prevalence threshold. *J Infect Dis* 2020; **221**: S503--S509.
39 Irvine MA, Reimer LJ, Njenga SM, *et al.* Modelling strategies to break transmission of lymphatic filariasis-aggregation, adherence and vector competence greatly alter elimination. *Parasit Vectors* 2015; **8**: 547.
40 Plaisier AP, Subramanian S, Das PK, *et al.* The LYMFASIM simulation program for modeling lymphatic filariasis and its control. *Methods Inf Med* 1998; **37**: 97–108.
41 Subramanian S, Stolk WA, Ramaiah KD, *et al.* The dynamics of Wuchereria bancrofti infection: a model-based analysis of longitudinal data from Pondicherry, India. *Parasitology* 2004; **128**: 467.
42 Stolk WA, Walker M, Coffeng LE, Basáñez M-G, de Vlas SJ. Required duration of mass ivermectin treatment for onchocerciasis elimination in Africa: a comparative modelling analysis. *Parasit Vectors*

2015; **8**: 552.
43 Stolk WA, De Vlas SJ, Borsboom GJJM, Habbema JDF. LYMFASIM, a simulation model for predicting the impact of lymphatic filariasis control: quantification for African villages. *Parasitology* 2008; **135**: 1583–98.
44 Stolk WA, Swaminathan S, Oortmarssen GJ van, Das PK, Habbema JDF. Prospects for elimination of bancroftian filariasis by mass drug treatment in Pondicherry, India: a simulation study. *J Infect Dis* 2003; **188**: 1371–81.
45 Stolk WA, de Vlas SJ, Habbema JDF. Anti-Wolbachia treatment for lymphatic filariasis. *Lancet* 2005; **365**: 2067.
46 Stolk WA, De Vlas SJ, Habbema JDF. Advances and challenges in predicting the impact of lymphatic filariasis elimination programmes by mathematical modelling. *Filaria J* 2006; **5**: 5.
47 Stolk WA, ten Bosch QA, de Vlas SJ, Fischer PU, Weil GJ, Goldman AS. Modeling the impact and costs of semiannual mass drug administration for accelerated elimination of lymphatic filariasis. *PLoS Negl Trop Dis* 2013; **7**: e1984.
48 Stolk WA, Stone C, de Vlas SJ. Modelling lymphatic filariasis transmission and control: modelling frameworks, lessons learned and future directions. *Adv Parasitol* 2015; **87**: 249–91.
49 Jambulingam P, Subramanian S, De Vlas SJ, Vinubala C, Stolk WA. Mathematical modelling of lymphatic filariasis elimination programmes in India: required duration of mass drug administration and post-treatment level of infection indicators. *Parasit Vectors* 2016; **9**: 501.
50 Irvine MA, Stolk WA, Smith ME, *et al.* Effectiveness of a triple-drug regimen for global elimination of lymphatic filariasis: a modelling study. *Lancet Infect Dis* 2017; **17**: 451–8.
51 Smith ME, Singh BK, Irvine MA, *et al.* Predicting lymphatic filariasis transmission and elimination dynamics using a multi-model ensemble framework. *Epidemics* 2017; **18**: 16–28.
52 Gambhir M, Michael E. Complex ecological dynamics and eradicability of the vector borne macroparasitic disease, lymphatic filariasis. *PLoS One* 2008; **3**: e2874.
53 Gambhir M, Bockarie M, Tisch D, *et al.* Geographic and ecologic heterogeneity in elimination thresholds for the major vector-borne helminthic disease, lymphatic filariasis. *BMC Biol* 2010; **8**: 22.
54 Michael E, Malecela-Lazaro MN, Simonsen PE, *et al.* Mathematical modelling and the control of lymphatic filariasis. *Lancet Infect Dis* 2004; **4**: 223–34.
55 Michael E, Malecela-Lazaro MN, Kabali C, Snow LC, Kazura JW. Mathematical models and lymphatic filariasis control: endpoints and optimal interventions. *Trends Parasitol* 2006; **22**: 226–33.
56 Singh BK, Bockarie MJ, Gambhir M, *et al.* Sequential modelling of the effects of mass drug treatments on anopheline-mediated lymphatic filariasis infection in Papua New Guinea. *PLoS One* 2013; **8**: e67004.
57 Singh BK, Michael E. Bayesian calibration of simulation models for supporting management of the elimination of the macroparasitic disease, lymphatic filariasis. *Parasit Vectors* 2015; **8**: 522.
58 Michael E, Singh BK. Heterogeneous dynamics, robustness/fragility trade-offs, and the eradication of the macroparasitic disease, lymphatic filariasis. *BMC Med* 2016; **14**: 14.
59 Verver S, Walker M, Kim YE, *et al.* How Can Onchocerciasis Elimination in Africa Be Accelerated? Modeling the Impact of Increased Ivermectin Treatment Frequency and Complementary Vector Control. *Clin Infect Dis* 2018; **66**: S267–74.
60 Plaisier AP, van Oortmarssen GJ, Habbema JD, Remme J, Alley ES. ONCHOSIM: a model and computer simulation program for the transmission and control of onchocerciasis. *Comput Methods Programs Biomed* 1990; **31**: 43–56.
61 Stolk WA, Walker M, Coffeng LE, Basáñez M-G, de Vlas SJ. Required duration of mass ivermectin treatment for onchocerciasis elimination in Africa: a comparative modelling analysis. *Parasit Vectors* 2015; **8**: 552.
62 Alley ES, Plaisier AP, Boatin BA, *et al.* The impact of five years of annual ivermectin treatment on skin microfilarial loads in the onchocerciasis focus of Asubende, Ghana. *Trans R Soc Trop Med Hyg* 1994; **88**: 581–4.
63 Coffeng LE, Stolk WA, Hoerauf A, *et al.* Elimination of African onchocerciasis: modeling the impact of increasing the frequency of ivermectin mass treatment. *PLoS One* 2014; **9**: e115886.

64 Duke BO. The population dynamics of Onchocerca volvulus in the human host. *Trop Med Parasitol* 1993; **44**: 61–8.
65 Plaisier AP. Modelling onchocerciasis transmission and control. 1996. http://repub.eur.nl/pub/21404/.
66 Habbema JDF, van Oortmarssen GJ, Plaisier AP. The ONCHOSIM model and its use in decision support for river blindness control. In: Isham V, Medley G, eds. Models for infectious human diseases. Their structure and relation to data. Cambridge, UK: Cambridge University Press, 1996: 360–80.
67 Plaisier AP, van Oortmarssen GJ, Remme J, Habbema JD. The reproductive lifespan of Onchocerca volvulus in West African savanna. *Acta Trop* 1991; **48**: 271–84.
68 Duke BO. Observations on Onchocerca volvulus in experimentally infected chimpanzees. *Tropenmed Parasitol* 1980; **31**: 41–54.
69 Prost A. Latence parasitaire dans I'onchocereose. *Bull World Heal Organ* 1980; **58**: 923–5.
70 Albiez EJ. Calcification in adult Onchocerca volvulus. *Trop Med Parasitol* 1985; **36**: 180–1.
71 Karam M, Schulz-Key H, Remme J. Population dynamics of Onchocerca volvulus after 7 to 8 years of vector control in West Africa. *Acta Trop* 1987; **44**: 445–57.
72 Schulz-Key H, Karam M. Periodic reproduction of Onchocerca volvulus. *Parasitol Today* 1986; **2**: 284–6.
73 Coffeng LE, Stolk WA, Zouré HGM, *et al.* African Programme for Onchocerciasis Control 1995-2015: model-estimated health impact and cost. *PLoS Negl Trop Dis* 2013; **7**: e2032.
74 Dadzie KY, Remme J, Rolland A, Thylefors B. The effect of 7-8 years of vector control on the evolution of ocular onchocerciasis in West African savanna. *Trop Med Parasitol* 1986; **37**: 263–70.
75 Kirkwood B, Smith P, Marshall T, Prost A. Relationships between mortality, visual acuity and microfilarial load in the area of the Onchocerciasis Control Programme. *Trans R Soc Trop Med Hyg* 1983; **77**: 862–8.
76 Prost A, Vaugelade J. La surmortalité des aveugles en zone de savane ouest-africaine. *Bull World Heal Organ* 1981; **59**: 773–6.
77 Plaisier AP, van Oortmarssen GJ, Remme J, Alley ES, Habbema JD. The risk and dynamics of onchocerciasis recrudescence after cessation of vector control. *Bull World Heal Organ* 1991; **69**: 169–78.
78 Philippon B. Etude de la transmission d' Onchocerca volvulus (Leuckart, 1983) Nematoda, Onchocercidae) par Simulium damnosum (Theobald, 1903) (Diptera, Simuliidae) en Afrique tropicale. *Trav Doc ORSTOM* 1977; **63**.
79 World Health Organization. Onchocerciasis Control Programme in West Africa: report of the annual OCP research meeting. 1989.
80 Plaisier AP, Alley ES, Boatin BA, *et al.* Irreversible effects of ivermectin on adult parasites in onchocerciasis patients in the Onchocerciasis Control Programme in West Africa. *J Infect Dis* 1995; **172**: 204–10.
81 Hamley JID, Milton P, Walker M, Basanez MG. Modelling exposure heterogeneity and density dependence in onchocerciasis using a novel individual-based transmission model, EPIONCHO-IBM: Implications for elimination and data needs. *PLoS Negl Trop Dis* 2019; **13**: e0007557.
82 Basanez MG, Boussinesq M. Population biology of human onchocerciasis. *Philos Trans R Soc L B Biol Sci* 1999; **354**: 809–26.
83 Filipe JA, Boussinesq M, Renz A, *et al.* Human infection patterns and heterogeneous exposure in river blindness. *Proc Natl Acad Sci U S A* 2005; **102**: 15265–70.
84 Lamberton PH, Cheke RA, Walker M, *et al.* Onchocerciasis transmission in Ghana: the human blood index of sibling species of the Simulium damnosum complex. *Parasit Vectors* 2016; **9**: 432.
85 Basanez MG, Collins RC, Porter CH, Little MP, Brandling-Bennett D. Transmission intensity and the patterns of Onchocerca volvulus infection in human communities. *Am J Trop Med Hyg* 2002; **67**: 669–79.
86 Prost A. Latence parasitaire dans l'onchocercose. *Bull World Heal Organ* 1980; **58**: 923–5.
87 Duke BOL. The effects of drugs on Onchocerca volvulus I. Methods of assessment, population dynamics of the parasite and the effects of diethylcarbamazine. *Bull World Heal Organ* 1968; **39**: 137–46.

88 Basanez MG, Walker M, Turner HC, Coffeng LE, de Vlas SJ, Stolk WA. River Blindness: Mathematical Models for Control and Elimination. *Adv Parasitol* 2016; **94**: 247–341.
89 Basanez MG, Pion SD, Boakes E, Filipe JA, Churcher TS, Boussinesq M. Effect of single-dose ivermectin on Onchocerca volvulus: a systematic review and meta-analysis. *Lancet Infect Dis* 2008; **8**: 310–22.
90 Eichner M. Onchocerca volvulus (Nematoda, Filarioidea) und Simulium damnosum-Komplex (Diptera): Die Entwicklung intrathorakal injizierter Mikrofilarien in verschiedenen Überträgerspecies Kameruns. 1989. http://epimos.com/index.php?id=141&L=1.
91 Dietz K. The population dynamics of onchocerciasis. In: Anderson RM, ed. Population Dynamics of Infectious Diseases. London: Chapman and Hall, 1982: 209–41.
92 Borlase A, Blumberg S, Callahan EK, *et al.* Modelling trachoma post 2020: Opportunities for mitigating the impact of COVID-19 and accelerating progress towards elimination. *Trans R Soc Trop Med Hyg*.
93 Pinsent A, Hollingsworth TD. Optimising sampling regimes and data collection to inform surveillance for trachoma control. *PLoS Negl Trop Dis* 2018; **12**: e0006531.
94 Pinsent A, Gambhir M. Improving our forecasts for trachoma elimination: What else do we need to know? *PLoS Negl Trop Dis* 2017; **11**: e0005378.
95 Grassly NC, Ward ME, Ferris S, Mabey DC, Bailey RL. The natural history of trachoma infection and disease in a Gambian cohort with frequent follow-up. *PLoS Negl Trop Dis* 2008; **2**: e341.
96 Shattock AJ, Gambhir M, Taylor HR, Cowling CS, Kaldor JM, Wilson DP. Control of trachoma in Australia: a model based evaluation of current interventions. *PLoS Negl Trop Dis* 2015; **9**: e0003474.
97 World Health Organization. Report of the 3rd Global Scientific Meeting on trachoma. 2010.
98 Liu F, Porco TC, Mkocha HA, *et al.* The efficacy of oral azithromycin in clearing ocular chlamydia: mathematical modeling from a community-randomized trachoma trial. *Epidemics* 2014; **6**: 10–7.
99 Bailey RL, Arullendran P, Whittle HC, Mabey DC. Randomised controlled trial of single-dose azithromycin in treatment of trachoma. *Lancet (London, England)* 1993; **342**: 453–6.
100 Bailey R, Duong T, Carpenter R, Whittle H, Mabey D. The duration of human ocular Chlamydia trachomatis infection is age dependent. *Epidemiol Infect* 1999; **123**: 479–86.
101 Le Rutte EA, Coffeng LE, Bontje DM, *et al.* Feasibility of eliminating visceral leishmaniasis from the Indian subcontinent: Explorations with a set of deterministic age-structured transmission models Quantitative analysis of strategies to achieve the 2020 goals for neglected tropical diseases: Wher. *Parasites and Vectors* 2016; **9**. DOI:10.1186/s13071-016-1292-0.
102 Le Rutte EA, Chapman LAC, Coffeng LE, *et al.* Elimination of visceral leishmaniasis in the Indian subcontinent: a comparison of predictions from three transmission models. *Epidemics* 2017; **18**: 67–80.
103 Le Rutte EA, Chapman LAC, Coffeng LE, *et al.* Policy Recommendations From Transmission Modeling for the Elimination of Visceral Leishmaniasis in the Indian Subcontinent. *Clin Infect Dis* 2018; **66**: S301–8.
104 Le Rutte EA, Zijlstra EE, de Vlas SJ. Post-Kala-Azar Dermal Leishmaniasis as a Reservoir for Visceral Leishmaniasis Transmission. *Trends Parasitol* 2019; **35**: 590–2.
105 Tiwary P, Kumar D, Mishra M, Singh RP, Rai M, Sundar S. Seasonal Variation in the Prevalence of Sand Flies Infected with Leishmania donovani. *PLoS One* 2013; **8**: e61370.
106 Poché D, Garlapati R, Ingenloff K, Remmers J, Poché R. Bionomics of phlebotomine sand flies from three villages in Bihar, India. *J Vector Ecol* 2011; **36**: 106–17.
107 Malaviya P, Hasker E, Picado A, *et al.* Exposure to phlebotomus argentipes (diptera, psychodidae, phlebotominae) sand flies in rural areas of Bihar, India: The role of housing conditions. *PLoS One* 2014; **9**: 1–7.
108 Picado A, Singh SP, Rijal S, *et al.* Longlasting insecticidal nets for prevention of Leishmania donovani infection in India and Nepal: paired cluster randomised trial. *BMJ* 2010; **341**: c6760.
109 Jervis S, Chapman LAC, Dwivedi S, *et al.* Variations in visceral leishmaniasis burden, mortality and the pathway to care within Bihar, India. *Parasit Vectors* 2017; **10**: 601.
110 Castaño MS, Ndeffo-Mbah ML, Rock KS, *et al.* Assessing the impact of aggregating disease stage data in model predictions of human African trypanosomiasis transmission and control activities in Bandundu province (DRC). *PLoS Negl Trop Dis* 2020; **14**: e0007976.

111 Crump RE, Huang C-I, Knock E, *et al.* Quantifying epidemiological drivers of gambiense human African Trypanosomiasis across the Democratic Republic of Congo. *medRxiv* 2020.
112 World Health Organisation. Number of new reported cases of human African trypanosomiasis (T.b. gambiense). 2018.
113 Stone CM, Chitnis N. Implications of heterogeneous biting exposure and animal hosts on Trypanosomiasis brucei gambiense transmission and control. *PLoS Comput Biol* 2015; **11**: e1004514.
114 Artzrouni M, Gouteux J-P. A compartmental model of sleeping sickness in central Africa. *J Biol Syst* 1996; **4**: 459–77.
115 Institut National de la Statistique, Ministère du Plan et Révolution de la modernité de la République Démocratique du Congo. Annuaire statistique 2014. 2015; : 69–71.
116 Rock KS, Ndeffo-Mbah ML, Castaño S, *et al.* Assessing strategies against Gambiense sleeping sickness through mathematical modeling. *Clin Infect Dis* 2018; **66**: S286--S292.
117 Rock KS, Torr SJ, Lumbala C, Keeling MJ. Quantitative evaluation of the strategy to eliminate human African trypanosomiasis in the Democratic Republic of Congo. *Parasit Vectors* 2015; **8**: 532.
118 Rock KS, Torr SJ, Lumbala C, Keeling MJ. Predicting the impact of intervention strategies for sleeping sickness in two high-endemicity health zones of the Democratic Republic of Congo. *PLoS Negl Trop Dis* 2017; **11**: e0005162.
119 Davis CN, Rock KS, Miaka EM, Keeling MJ. Village-scale persistence and elimination of gambiense human African trypanosomiasis. *PLoS Negl Trop Dis* 2019; **13**: 1–15.
120 OCHA Office for the Coordination of Humanitarian Affairs. Journées Nationales de Vaccination (JNV) Activités de vaccination supplementaire , RDC. .
121 The World Bank. Data:Democratic Republic of Congo. http://data.worldbank.org/country/congo-democratic-republic.
122 Rogers DJ. A general model for the African trypanosomiases. *Parasitology* 1988; **97 (Pt 1)**: 193–212.
123 Checchi F, Filipe JAN, Barrett MP, Chandramohan D. The natural progression of Gambiense sleeping sickness: what is the evidence? *PLoS Negl Trop Dis* 2008; **2**: e303.
124 Checchi F, Funk S, Chandramohan D, Haydon DT, Chappuis F. Updated estimate of the duration of the meningo-encephalitic stage in gambiense human African trypanosomiasis. *BMC Res Notes* 2015; **8**: 292.
125 Mpanya A, Hendrickx D, Vuna M, *et al.* Should I get screened for sleeping sickness? A qualitative study in Kasai province, Democratic Republic of Congo. *PLoS Negl Trop Dis* 2012; **6**: e1467.
126 Checchi F, Chappuis F, Karunakara U, Priotto G, Chandramohan D. Accuracy of Five Algorithms to Diagnose Gambiense Human African Trypanosomiasis. *PLoS Negl Trop Dis* 2011; **5**: e1233.
127 Davis S, Aksoy S, Galvani A. A global sensitivity analysis for African sleeping sickness. *Parasitology* 2011; **138**: 516–26.
128 Ravel S, Grébaut P, Cuisance D, Cuny G. Monitoring the developmental status of Trypanosoma brucei gambiense in the tsetse fly by means of PCR analysis of anal and saliva drops. *Acta Trop* 2003; **88**: 161–5.
129 WHO. Control and surveillance of human African trypanosomiasis. 2013.
130 Clausen PH, Adeyemi I, Bauer B, Breloeer M, Salchow F, Staak C. Host preferences of tsetse (Diptera: Glossinidae) based on bloodmeal identifications. *Med Vet Entomol* 1998; **12**: 169–80.
